# Agricultural activities and risk of treatment for depressive disorders among the entire French agricultural workforce: the TRACTOR project, a nationwide retrospective cohort study

**DOI:** 10.1016/j.lanepe.2023.100674

**Published:** 2023-06-26

**Authors:** Pascal Petit, Gérald Gandon, Marc Dubuc, Nicolas Vuillerme, Vincent Bonneterre

**Affiliations:** a Univ. Grenoble Alpes, CNRS, UMR 5525, VetAgro Sup, Grenoble INP, CHU Grenoble Alpes, TIMC, 38000 Grenoble, France; b CHU Grenoble Alpes, Centre Régional de Pathologies Professionnelles et Environnementales, 38000 Grenoble, France; c Univ. Grenoble Alpes, AGEIS, 38000 Grenoble, France; d CHU Grenoble Alpes, Service de psychiatrie (psychiatrie de liaison/VigilanS), 38000 Grenoble, France; e Institut Universitaire de France, Paris, France

**Keywords:** Administrative health database, Agriculture, Farming, Depression, Depressive disorders, Mental health, Health surveillance, Epidemiology, Data mining, Europe, France

## Abstract

**Background:**

Although depression is a major issue among farming population, to date, there have been few studies on specific agricultural activities. We aimed to investigate whether, among the entire French farm manager (FM) workforce, certain agricultural activities are more strongly associated with depression than others.

**Methods:**

This nationwide retrospective cohort study used data from an administrative health database available to the TRACTOR project. This database pertains to the entire French agricultural workforce (overseas workers not included). Data were analyzed from January 2021 to December 2022. All FMs that worked at least once over the period 2002–2016 were included. The outcome measure was the association between 26 agricultural activities and the risk of depression measured as hazard ratios (HRs) after adjusting for age, sex, and pre-existing medical comorbidities. The time to first depression insurance declaration, or first antidepressant prescription claim was used as the underlying timescale. For each activity, the reference/control group included all FMs that never performed the considered activity between 2002 and 2016, while the exposed group included FMs that performed the considered activity at least once from 2002 to 2016. Four sensitivity analyses were conducted to test hypotheses, and to address potential sources of bias.

**Findings:**

There were 84,507 (7.76%; 28.2 cases per 1000 person-years) depression cases among 1,088,561 FMs (mean age 46.6 [SD 14.1]). Compared to other activities, dairy farming (HR = 1.37, 95% confidence interval: 1.32–1.42), cow farming (HR = 1.53 [1.47–1.59]), poultry and rabbit farming (HR = 1.37 [1.27–1.50]), and mixed farming (HR = 1.30 [1.24–1.36]) were more strongly associated with depression. Sex differences were observed, with most of the time, risks higher for females than for males.

**Interpretation:**

Agricultural activities at risk of depression among the entire French agricultural workforce were identified. These findings do represent a crucial first step on the road to implement effective preventive measures against depression to determine where additional resources should be allocated to screen for depression, along with intervention.

**Funding:**

MIAI@Grenoble Alpes, and 10.13039/501100009424Mutualité Sociale Agricole.

**Research in context**

**Evidence before this study**

PubMed was searched on December 15, 2022, with no restrictions on language or publication date, using the following search strategy: “depression AND (agriculture OR farm OR farming) AND (work OR occupation OR activity OR task)”. Most studies that investigated the risk of depression among the farming population were often limited in size and scope and examined either the risk of depression for overall agriculture or for few specific activities, mainly dairy and crop farming. In France, only two case–control studies and two longitudinal cohorts were identified, which pertained to less than 2200 farmers. Further longitudinal research involving entire population samples is needed. Administrative data are increasingly used to conduct research on depression and inform health services, and health policy. No study using nationwide administrative health data to examine the risk of depression in farmer was found.

**Added value of this study**

To the best of our knowledge, this is the first study using data from insurance health databases pertaining to the entire French farm manager population (overseas workers not included) that investigated the associations between 26 agricultural activities and depression, overall, and by sex category. Increased risks of depression were observed for several activities. Cow farming, poultry and rabbit farming, dairy farming, and mixed farming were the activities the most at risk of depression within the farm manager population.

**Implications of all the available evidence**

Depression surveillance using administrative health data is an alternative and a complementary approach to traditional cohort studies, which can be more resource-intensive. Findings from this study will be of interest to policy makers and public health practitioners because they can help to determine potential agricultural activities for which further studies could be needed. Additional works are required to identify risk factors, and to determine whether there is a need to implement a universal public health surveillance in the French farm manager population, along with precision interventions in specific activities.

## Introduction

Farmers are an understudied population for which mental health issues is a public health concern.^1^, ^2^, ^3^ Farmers have to deal with many factors outside their control, such as weather condition, government regulation, and market volatility.^3^^,^^4^ In addition to these factors, physical demands and economic woes intertwine with a personal responsibility for land that often is passed down through generations. These factors represent some of the major stressors that can lead to mental health issues such as depression.

Depression is one of the most common mental disorders, affecting around 280 million people worldwide, and accounting for more than 47 million disability-adjusted life years in 2019.^5^ Depression presents the largest mental health disease burden in higher-income countries, and is the second leading cause of life years spent with disability, contributing to a significant loss of productivity.^6^, ^7^, ^8^, ^9^, ^10^

Different types of agriculture (e.g., animal husbandry, crop farming) may be associated with differing work-related characteristics, which could potentially relate to depression. Sex differences could also exist.^3^ Identifying specific and common/shared risk factors between agricultural practices, overall and by sex category, is paramount for implementing effective preventive measures, and essential for reducing the burden of depression.^4^ However, despite a large number of studies investigating depression among farmers worldwide,^1^^,^^8^^,^^11^, ^12^, ^13^ most studies are often limited in size and scope, do not usually describe specific farming subpopulation other than migrant workers, and do not usually investigate sex differences.^1^^,^^3^ In addition, existing works also examined either the risk of depression for overall agriculture or for few specific activities, and are mostly cross-sectional studies.^1^^,^^3^ In France, only two case–control studies and two longitudinal cohorts pertaining to less than 2200 farmers, and to restricted geographical areas were conducted.^14^, ^15^, ^16^, ^17^ None of these four studies investigated the risk of depression related to specific agricultural activities. Further longitudinal research involving entire population samples is needed. Administrative data are increasingly used to conduct research on depression and inform health services, and health policy.^7^

This study investigates whether, among the entire French farm manager (FM) population, certain agricultural activities are more strongly associated with depression than other activities, overall and by sex category.

## Methods

### Data source, and study population

All FMs within the TRACTOR project, including farm or company managers, owners, and self-employed persons, living and working in mainland France (urban and rural areas) were included in this study if they worked at least one year from 2002 to 2016. The TRACTOR project, and the study population were described previously.^18^ Briefly, sociodemographic characteristics are routinely collected each year by Mutualité Sociale Agricole (MSA) from forms that are filled by FMs during their yearly insurance affiliation. Each occupational activity is then coded by MSA according to an internal thesaurus referring to 26 different agricultural activities (Table S1). These activities refer to the main activity in terms of effective working time during a year. Health data from 2012 to 2016 were used as follow-up period, with January 1st 2012 as the baseline time point (i.e., time zero), and December 31st 2016 as the follow-up end. Health data pertained to drug prescription, and to chronic diseases/long-term illnesses (LTIs) for which FMs are entitled to fee exemption, and the full coverage of health care expenditures. There was no missing data regarding the variables of interest for this study.

Data were obtained and managed from October 2018 to December 2020,^18^ and analyzed from January 2021 to December 2022. Administrative health data were not available after 2016. The French independent administrative authority protecting privacy and personal data approved this study. All methods were performed in accordance with the relevant guidelines and regulations. As MSA provided data after encryption to protect private information, the need for informed consent was waived. This study followed the Strengthening the Reporting of Observational Studies in Epidemiology (STROBE) reporting guideline (available in the supplementary material).

### Choice of primary measure

A review suggested that the best algorithm to identify depression cases using administrative data consists in including both international classification of disease codes, and antidepressant (ATD) prescriptions.^10^ We followed this approach by identifying depression cases using both ICD-10 codes (10th revision of the International Statistical Classification of Diseases and Related Health Problems), and ATC codes (Anatomical Therapeutic Chemical Classification System) related to depression. ICD-10 codes F32 (“Depressive episode”) and F33 (“Major depressive disorder, recurrent”) from LTIs, as well as all ATC codes related to all types of ATD prescriptions (N06A) were used as a proxy for the treatment of depression and depressive symptoms.

To the authors’ knowledge, there is no gold standard regarding the number of prescriptions to use for identifying depression cases.^10^^,^^19^ Therefore, we considered 13 algorithms: at least one LTI declaration for depression (F32 or F33), and from one to 12 ATD prescriptions. For these analyses, we considered that depression cases could be work-related only among FMs that had a depression insurance declaration (LTI), or ATD prescription claim after the start of their activity, but no more than two years after the end of their activity (Fig. S1).

### Statistical analysis

To determine whether certain agricultural activities are more strongly associated with depression than other activities, hazard ratios (HRs) and 95% confidence intervals were estimated using Cox proportional hazards model, with time to first depression insurance declaration, or ATD drug prescription as the underlying timescale. The dependent variables of the model were the timescale (continuous), and the depression diagnosis (two categories: yes or no). One model was created for each activity. The assumption of proportional hazard rate were checked for each model by verifying the independence of scaled Schoenfeld's residuals and time. The median follow-up was estimated using the Kaplan–Meier reverse approach.

As we did not have access to the general population nor to other occupational sectors such as banking or administration, the reference/control group included all FMs who did not carry out the activity of interest, while the exposed group included all FMs that performed the activity of interest. For instance, for crop farmers, the reference group included all FMs that never farmed crops between 2002 and 2016, while the exposed group included FMs that were crop farmers at least once from 2002 to 2016. For dairy farming, the reference group included all FMs that were never dairy farmers between 2002 and 2016, while the exposed group included FMs that were dairy farmers at least once from 2002 to 2016.

Some FMs performed more than one activity over the duration of the study (e.g., pig farmers between 2002 and 2008, and then poultry farmers between 2009 and 2016), which lead to the existence of overlap in farming activities. As a result, 26 separate models (one for each activity) had to be created. Depression risks were estimated according to each of the 26 activities when there were at least three exposed cases. For each activity, 13 analyses (one for each algorithm) were conducted. An aggregated/consensual risk estimate (HR) was then calculated based on a linear combination of HR risk distributions that were obtained from one to 13 algorithms depending on the number of exposed cases available. The construction of the aggregated risk estimate was based on the weighted linear aggregation (arithmetic mean) of the probability risk distribution (i.e., HR distribution) generated using a given risk estimate (HR). The weights were calculated using the degree of agreement among the risk estimate-based risk distributions.^20^ Results for the consensual risk estimates are presented in the main text and results for each algorithm, and all sensitivity analyses are available in the supplementary material (Tables S2–S7). All analyses were adjusted for age, sex (only when considering both sexes), first year of the farm's establishment, number of working years, and number of pre-existing medical comorbidities (Table 1). Pre-existing medical comorbidities were defined as the number of LTIs (e.g., baseline/pre-existing levels of mental disorders, chronic diseases such as diabetes, cancers) that were declared before the first LTI declaration for depression, or the first ATD prescription. As sex is a risk factor for depression, interaction tests were conducted to statistically evaluate sex differences by adding interaction terms in the model. Sex specific analyses (subgroup analyses), with one separate model for each sex, were also conducted to determine sex-specific risk estimates. The Benjamini-Hochberg approach was used to account for multiple testing.

**Table 1:** List of variables considered in the analyses.

Dependent variable	Modality	Analysis
Depression diagnosis (LTI declaration or ATD prescription)	2 categories: yes or no	MA, SA1, SA2, SA3, SA4
Time to first depression insurance declaration, or ATD drug prescription	continuous	MA, SA1, SA2, SA3, SA4
**Independent variables – covariates always included**		
Activity	2 categories: yes or no	MA, SA1, SA2, SA3, SA4
Number of years performing the considered activity	continuous	MA, SA1, SA2, SA3, SA4
First year of the farm's establishment	continuous	MA, SA1, SA2, SA3, SA4
Sex^a^	2 categories: female or male	MA, SA1, SA2, SA3, SA4
Age	continuous	MA, SA1, SA2, SA3, SA4
Number of pre-existing medical comorbidities	continuous	MA, SA2, SA3, SA4
**Independent variables – potential covariates selected based on VIF**		
Family status	2 categories: single or as a couple	SA3
Median farm surface	continuous	SA3
Farm location	96 categories: administrative departments	SA3
Number of farms	2 categories: 1 farm or > 1 farm	SA3
Farm type (farm clustering)	2 categories: individual farm or farm with work partners	SA3
Partner work status	2 categories: do not perform task to help farm manager or perform task to help farm manager	SA3
Number of associates	2 categories: 0 or ≥ 1	SA3
Secondary activity	2 categories: no secondary activity or at least one secondary activity	SA3
Lack of job security	2 categories: has never been unemployed during the observation period or has been unemployed during the observation period	SA3
Median insurance premium	continuous	SA3
Employees	2 categories: no employee or at least one employee	SA3
Pre-existing disability	2 categories: did not become disabled before the disease of interest declaration or became disabled before the disease of interest declaration	SA3
Working years	15 categories: 2002, 2003 … 2016	SA3

Note: MA: main analysis, SA1: first sensitivity analysis, SA2: second sensitivity analysis, SA3: third sensitivity analysis, SA4: fourth sensitivity analysis, VIF: variance inflation factor.

a The analysis was adjusted on sex only for “both sexes”; otherwise, the sex was used to conduct subgroup analyses.

Four sensitivity analyses were conducted to test hypotheses, and to address potential sources of bias. Since pre-existing medical comorbidities could be on the causal pathway from exposure to outcome and cause bias in adjustments, a sensitivity analysis excluding these confounders was performed (SA1). To assess potential bias resulting from not considering depression as potentially work-related if the diagnosis occurred more than two years after the end of an activity, a sensitivity analysis (SA2) that considered that all depression cases could be work-related, regardless of the date of diagnosis (Fig. S1), was conducted. The third sensitivity analysis (SA3) adjusted on the same covariates than the main analysis in addition to all other potential cofounders available (e.g., secondary activities, farm surface, family status), that were selected based on the variance inflation factor (VIF) (VIF≤ 2.5) to remove collinear variables. To strengthen the assumption that only mental health problems were included, a sensitivity analysis (SA4) was restricted to selective serotonin reuptake inhibitors (SSRIs) (Table 1).

All statistical analyses were performed using R software 4.1.2® (R Core Team, Vienna, Austria) for Windows 10©.

### Role of the funding source

This work was partially supported by MIAI@Grenoble Alpes [ANR-19-P3IA-0003, 2019], and was supported within the STOP project (Searching and Tracking Occupational factors to Prevent suicide in agriculture) by MSA [MSA-2020-STOP, 2020]. The funding sources had no role in the study design and conduct of the study; in the collection, management, analysis, and interpretation of data; in the writing of the report; preparation, review, or approval of the article; or in the decision to submit the paper for publication. The authors were not precluded from accessing data in the study, and all authors took final responsibility in the decision to submit for publication.

## Results

### Population characteristics

Baseline characteristics of the study population are presented in Table 2. Among the 1,088,561 FMs available to TRACTOR over the period 2002–2016, 84,507 cases (7.76%) were identified during the follow-up period. The median follow-up was 1460 (1096; 1825) days. Overall, there were 2,995,264 person-years, with 794,862 person-years for female, and 2,200,402 person-years for male, respectively. There were 28.2 [28.0–28.4] cases per 1000 person-years, with 46.6 [46.1–47.1] cases per 1000 person-years for female, and 21.6 [21.4–21.8] cases per 1000 person-years for male, respectively. The proportion of female was higher for FMs with a depression than without (44% *vs.* 30%). Overall, FMs with a depression were older than FMs without a depression (49.1 [SD 13.4] years old *vs.* 46.4 [SD 14.2]), established their farm in earlier time periods, were more often in a couple (64% *vs.* 56%), had more often employees (33% *vs.* 27%), had bigger farm surfaces, paid higher insurance premiums, had a higher number of pre-existing medical comorbidities (42% *vs.* 24%), and performed less often a secondary activity (17% *vs.* 39%). No information on sibship/siblings, or couples was available.

**Table 2:** Characteristics of the study population, TRACTOR project, France, 2002–2016.

Main characteristics	FM without depression (n = 1,004,054)	FM with depression (n = 84,507)
	n (%)	n (%)
**Sex**		
Female	300,218 (30)	37,045 (44)
Male	703,836 (70)	47,462 (56)
**Age (years)**		
Mean (SD)	46.4 (14.2)	49.1 (13.4)
**Family status**		
Single	438,382 (44)	30,628 (36)
As a couple	565,672 (56)	53,879 (64)
**First year of the farm's establishment**		
Median (IQR)	1994 (17)	1992 (14)
**Farm surface (expressed in hectares)**		
Median (IQR)	15.7 (4.4)	25.1 (5.5)
**Farm location (region)**		
Auvergne-Rhône-Alpes	108,850 (10.8)	8578 (10.2)
Bourgogne-Franche-Comté	60,456 (6.0)	5408 (6.4)
Bretagne	74,719 (7.4)	6583 (7.8)
Centre - Val de Loire	45,569 (4.5)	4265 (5.1)
Corse	4949 (0.5)	377 (0.4)
Grand Est	75,520 (7.5)	6234 (7.4)
Hauts-de-France	44,472 (4.4)	3926 (4.7)
Île-de-France	12,955 (1.3)	1129 (1.3)
Normandie	73,899 (7.4)	6739 (8.0)
Nouvelle-Aquitaine	165,248 (16.5)	15,135 (17.9)
Occitanie	155,051 (15.4)	11,774 (13.9)
Provence-Alpes-Côte d'Azur	105,964 (10.6)	8089 (9.6)
Pays de la Loire	76,402 (7.6)	6270 (7.4)
**Number of farms**		
1 farm/>1 farm	980,011 (98)/24,043 (2)	82,099 (97)/2408 (3)
**Farm type (farm clustering)**		
Individual farm	700,989 (70)	55,599 (66)
Farm with work partners	303,065 (30)	28,908 (34)
**Partner work status**		
Do not perform task to help farm manager	887,853 (88)	72,457 (86)
Perform task to help farm manager	116,201 (12)	12,050 (14)
**Number of associates**		
0/≥ 1	774,790 (77)/229,264 (23)	61,442 (73)/23,065 (27)
**Secondary activity**		
No secondary activity	616,535 (61)	70,055 (83)
At least one secondary activity	387,519 (39)	14,452 (17)
**Lack of job security**		
Has never been unemployed during the observation period	1,002,149 (99.8)	84,294 (99.7)
Has been unemployed during the observation period	1905 (0.2)	213 (0.3)
**Median yearly insurance premium (euros)**		
Median (IQR)	5064 (9987)	6126 (10,612)
**Employees**		
No employee	733,262 (73)	56,882 (67)
At least one employee	270,792 (27)	27,625 (33)
**Specialist consult**		
No	347,962 (35)	0 (0)
Yes	656,092 (65)	84,507 (100)
**GP consult**		
No	362,178 (36)	222 (0.3)
Yes	641,876 (64)	84,285 (99.7)
**Pre-existing disability**		
Did not become disabled before the disease of interest declaration	1,000,549 (99.6)	83,593 (99)
Became disabled before the disease of interest declaration	3505 (0.3)	914 (1)
**Number of pre-existing comorbidities (LTI)**		
0 comorbidity before the disease of interest declaration	761,360 (76)	48,685 (58)
1 comorbidity before the disease of interest declaration	123,556 (12)	16,347 (19)
>1 comorbidity before the disease of interest declaration	119,138 (12)	19,475 (23)

Note: FM: farm manager, GP: general practitioner, IQR: interquartile range, LTI: long-term illness, SD: arithmetic standard deviation.

Most depression cases were identified with ATD prescription claims (Fig. S2). From 2012 to 2016, there were 83,592 FMs that had at least one ATD prescription, 5072 FMs with a LTI for depression, including 915 (18%) that had no ATD prescription. Nearly one-fourth (24.6%) had only one ATD prescription, while 28% had at least 12 ATD prescriptions, which means they were under ATD treatment for more than 12 months. Most FMs (64.6%) had a depression insurance declaration, or ATD prescription claim between 2012, and no more than two years after the end of their activity (Fig. S3). Most ATDs prescribed were SSRIs (64.6%) (Fig. S4). Most FMs were prescribed with only one ATD agent (74.5%), while 17.5% were prescribed with two, and 8% with more than two (Fig. S5).

### Depression risk associated with agricultural activities

For all models, the assumption of proportional hazard rate was met for each covariate. Associations varied by sex and types of crops, and animal farming (Table 3, Table S3). Table S3 presents the results of the interaction tests conducted to assess sex differences. For other tables (Table 3, and Tables S4–S7), results for both sexes (analysis adjusted on sex) along with subgroup results (one separate model for each sex) are presented.

**Table 3:** Agricultural activities and risks of depression, TRACTOR, France, 2002–2016 - results from the main analysis.

Activity	Both sexes (n = 1,088,561)					Female (n = 337,263)					Male (n = 751,298)				
	n (%)	m (range)	HR	p	p.adj	n (%)	m (range)	HR	p	p.adj	n (%)	m (range)	HR	p	p.adj
Truck farming, floriculture/flower-growing	43,487 (3.99)	80; 1855	1.06 [0.98–1.14]	0.19	0.25	13,251 (3.93)	26; 728	1.10 [0.99–1.23]	0.14	0.17	30,236 (4.02)	54; 1127	1.04 [0.93–1.16]	0.57	0.82
Fruit arboriculture	25,001 (2.3)	51; 1027	0.99 [0.90–1.10]	0.73	0.96	7927 (2.35)	21; 405	0.87 [0.74–1.04]	0.15	0.20	17,074 (2.27)	30; 622	1.08 [0.94–1.23]	0.26	0.41
Garden center/tree nursery	5380 (0.49)	11; 282	1.30 [1.07–1.60]	0.05	0.06	1419 (0.42)	4; 95	1.35 [0.98–1.90]	0.13	0.15	3961 (0.53)	7; 187	1.28 [1.00–1.67]	0.09	0.12
Crop farming (e.g., wheat, corn, and industrial grower)	316,926 (29.1)	547; 10,755	**0.90 [0.87–0.93]**	3.1e-4	**3.4e-4**	105,642 (31.3)	261; 4272	**0.88 [0.83–0.93]**	0.02	**0.02**	211,284 (28.1)	286; 6483	0.95 [0.91–0.99]	0.05	0.07
Viticulture	121,950 (11.2)	266; 6531	**1.09 [1.05–1.13]**	8.8e-4	**9.1e-4**	42,909 (12.7)	112; 2825	1.06 [1.00–1.12]	0.08	0.12	79,041 (10.5)	154; 3706	**1.12 [1.06–1.19]**	0.03	**0.03**
Sylviculture/forestry (e.g., thinning, pruning)	2160 (0.2)	6; 80	1.42 [1.01–2.01]	0.07	0.09	359 (0.11)	4; 19	1.39 [0.75–2.60]	0.32	0.51	1801 (0.24)	2; 61	1.62 [1.09–2.41]	0.04	0.05
Unspecified specialized farming (e.g., herbs, mushrooms)	6615 (0.61)	22; 271	**1.36 [1.14–1.63]**	1.1e-3	**1.4e-3**	2408 (0.71)	11; 122	1.23 [0.95–1.60]	0.12	0.17	4207 (0.56)	11; 149	**1.42 [1.11–1.83]**	8.7e-3	**0.01**
Dairy farming	161,436 (14.8)	545; 9135	**1.37 [1.32–1.42]**	<0.0001	**<0.0001**	49,659 (14.7)	228; 3615	**1.41 [1.33–1.48]**	<0.0001	**<0.0001**	111,777 (14.9)	317; 5520	**1.27 [1.21–1.33]**	<0.0001	**<0.0001**
Cow farming	111,873 (10.3)	481; 6933	**1.53 [1.47–1.59]**	<0.0001	**<0.0001**	32,904 (9.76)	203; 2840	**1.64 [1.56–1.74]**	<0.0001	**<0.0001**	78,969 (10.5)	278; 4093	**1.41 [1.34–1.49]**	<0.0001	**<0.0001**
Both/mixed dairy and cow farming	31,070 (2.85)	98; 1834	**1.21 [1.12–1.30]**	7.0e-4	**7.3e-4**	8084 (2.4)	37; 646	**1.31 [1.17–1.48]**	3.3e-3	**3.7e-3**	22,986 (3.06)	61; 1188	1.10 [1.01–1.22]	0.05	0.08
Ovine and caprine farming	48,716 (4.48)	150; 2327	**1.11 [1.04–1.18]**	3.5e-3	**4.1e-3**	17,314 (5.13)	70; 1093	1.11 [1.02–1.22]	0.05	0.06	31,402 (4.18)	80; 1234	1.10 [1.01–1.20]	0.03	**0.05**
Pig farming	13,636 (1.25)	42; 845	**1.27 [1.14–1.43]**	6.2e-3	**6.4e-3**	3890 (1.15)	20; 345	**1.49 [1.26–1.77]**	7.6e-4	**7.7e-4**	9746 (1.3)	22; 500	1.12 [0.97–1.30]	0.16	0.22
Stud farming	17,164 (1.58)	33; 671	1.09 [0.95–1.25]	0.38	0.49	7408 (2.2)	20; 402	0.99 [0.83–1.18]	0.55	0.79	9756 (1.3)	13; 269	1.24 [1.00–1.54]	0.08	0.11
Training, dressage, riding clubs	14,874 (1.37)	19; 742	0.98 [0.84–1.15]	0.40	0.53	6619 (1.96)	9; 411	0.97 [0.80–1.18]	0.43	0.57	8255 (1.1)	10; 331	1.00 [0.79–1.27]	0.62	0.90
Unspecified large animal farming (e.g., ostrich, llama)	2997 (0.28)	5; 149	**1.96 [1.53–2.53]**	0.02	**0.02**	1437 (0.43)	3; 93	**1.73 [1.27–2.42]**	0.03	**0.03**	1560 (0.21)	2; 56	**1.98 [1.32–2.98]**	0.02	**0.02**
Poultry and rabbit farming	25,540 (2.35)	81; 1620	**1.37 [1.27–1.50]**	<0.0001	**<0.0001**	9995 (2.96)	39; 824	**1.40 [1.25–1.57]**	3.6e-3	**3.8e-3**	15,545 (2.07)	42; 796	**1.35 [1.20–1.51]**	4.0e-4	**4.3e-4**
Unspecified small animal farming (e.g., frogs, snails, bees)	19,896 (1.83)	31; 614	0.87 [0.75–1.00]	0.08	0.09	8596 (2.55)	10; 277	**0.58 [0.47–0.72]**	3.7e-3	**4.1e-3**	11,300 (1.5)	21; 337	1.21 [1.02–1.44]	0.03	0.05
Shellfish farming (e.g., oyster farming, scallop aquaculture)	3795 (0.35)	7; 159	**1.40 [1.08–1.84]**	0.04	**0.05**	725 (0.22)	2; 45	1.31 [0.86–2.01]	0.25	0.37	3070 (0.41)	5; 114	**1.65 [1.20–2.30]**	0.02	**0.02**
Unspecified and mixed farming (e.g., polyculture, mixed farming, diversified farming)	127,900 (11.7)	346; 6690	**1.30 [1.24–1.36]**	<0.0001	**<0.0001**	39,338 (11.7)	144; 2679	**1.35 [1.26–1.44]**	<0.0001	**<0.0001**	88,562 (11.8)	202; 4011	**1.21 [1.14–1.28]**	1.3e-4	**1.3e-4**
Salt works/salt evaporation pond	974 (0.09)	2; 40	0.92 [0.57–1.50]	0.74	0.96	221 (22.7)	0; 3	0.37 [0.15–0.96]	0.04	0.05	753 (0.1)	2; 34	1.40 [0.85–2.34]	0.20	0.32
Wood production (e.g., lopping)	11,385 (1.05)	13; 504	1.13 [0.95–1.34]	0.19	0.26	297 (0.09)	0; 22	**2.13 [1.18–3.87]**	0.02	**0.03**	11,088 (1.48)	13; 482	1.17 [0.98–1.42]	0.13	0.16
Stationary sawmill (e.g., edging, trimming, decking, debarking)	787 (0.07)	3; 45	1.59 [1.00–2.65]	0.14	0.17	52 (0.02)	0; 52	**3.27 [1.31–8.43]**	0.03	**0.04**	735 (0.1)	3; 37	1.45 [0.87–2.47]	0.26	0.33
Agricultural work companies (e.g., pesticide applications, harvest reaping)	15,557 (1.43)	20; 656	1.19 [1.02–1.38]	0.09	0.11	1866 (0.55)	3; 107	1.40 [1.03–1.94]	0.07	0.08	13,691 (1.82)	17; 549	1.18 [1.00–1.41]	0.15	0.17
Gardening, landscaping and reforestation companies	48,878 (4.49)	76; 2309	**1.30 [1.19–1.43]**	0.01	**0.01**	2531 (0.75)	9; 169	**1.62 [1.25–2.12]**	7.4e-3	**8.3e-3**	46,347 (6.17)	67; 2140	**1.49 [1.36–1.63]**	2.2e-3	**2.5e-3**
Company representative/authorized representative	1931 (0.18)	2; 42	0.91 [0.60–1.39]	0.65	0.94	1496 (0.44)	1; 35	0.84 [0.53–1.32]	0.45	0.69	437 (0.06)	1; 7	1.26 [0.45–3.64]	0.65	0.91
Rural craftsperson (e.g., mason, mechanics)	7701 (0.71)	0; 5	**0.03 [0.01–0.08]**	<0.0001	**<0.0001**	283 (0.08)	0	NC	NC	NC	7418 (0.99)	0; 5	**0.03 [0.01–0.08]**	<0.001	**<0.001**

Note: HR: hazard ratio, n: number of exposed farm managers, m: range of the number of exposed depression cases, NC: not calculated, p: p-value, p.adj: p-value adjusted using the Benjamini-Hochberg approach. Hazard ratios were estimated by Cox models with time to first depression insurance declaration or first antidepressant prescription as the underlying timescale, when the number of exposed cases was sufficient (m ≥ 3), adjusted for sex (both sexes only), age, first year of the farm's establishment, number of working years, and number of pre-existing medical comorbidities. Results for females and males were obtained with subgroup analysis (one separate model for each sex).

For both sexes, compared to other agricultural activities, increased risks of depression were found for all activities involving cattle, for poultry and rabbit farming (HR = 1.37 [1.27–1.50]), unspecified large animal farming (HR = 1.96 [1.53–2.53]), unspecified and mixed farming (HR = 1.30 [1.24–1.36]), and gardening, landscaping and reforestation companies (HR = 1.30 [1.19–1.43]) (Table 3). Sex differences were found for 12 out of 26 activities (Table S3), with risks higher for females than males, with the exception of crop farming, unspecified small animal farming, unspecified and mixed farming, training, dressage, riding clubs, and gardening, landscaping and reforestation companies.

Regarding females, pig farming (HR = 1.49 [1.26–1.77]), wood production (HR = 2.13 [1.18–3.87]), and stationary sawmill (HR = 3.27 [1.31–8.43]) were found with increased risk of depression, while crop farming (HR = 0.88 [0.83–0.93]), and unspecified small animal farming (HR = 0.58 [0.47–0.72]) were found with decreased risks compared to other agricultural activities.

Regarding males, increased risks of depression were observed for viticulture (HR = 1.12 [1.06–1.19]), unspecified specialized farming (HR = 1.42 [1.11–1.83]), and shellfish farming (HR = 1.65 [1.20–2.30]). By contrast, rural craftsperson (HR = 0.03 [0.01–0.08]) were found with decreased risk of depression, but the number of exposed cases was low.

SA1, which did not adjust on pre-existing medical comorbidities, yielded similar results than the main analysis (Table S4). The only exception was observed for fruit arboriculture that was found with decreased risks in females. There were ten activities with sex differences, with three (crop farming, unspecified small animal farming, and salt works/salt evaporation pond) for which risks were higher for males than females (Table S3).

SA2, which considered that depression could be work-related regardless of its time of diagnosis after the start of an activity (Fig. S1), yielded mostly similar results than the main analysis (Table S5). There were 16 activities that exhibited sex differences, with most of the time (n = 10), risks higher for females than for males. Contrary to the main analysis, SA2 yielded increased risks in males for crop farming, pig farming, and company representatives, as well as decreased risks in males for activities involving horses. There were also no increased risks observed for females in wood production, stationary sawmill, and unspecified small animal farming, and for males in shellfish farming, mixed farming, unspecified large animal farming, and unspecified specialized farming.

SA3, which adjusted on the same covariates than the main analysis in addition to all other potential cofounders available (Table 1), yielded similar results than the main analysis (Table S6). The only exceptions were observed for truck farming that was found with decreased risks in both females and males, and with agricultural work companies that was found with increased risk in females only. There were 12 activities our of 26 that were found with sex differences, with risks higher for females than for males, except for crop farming, unspecified small animal farming, and salt works/salt evaporation pond.

SA4, which was restricted to SSRIs, yielded similar results than the main analysis (Table S7). The only exceptions were observed in males, with increased risks in ovine and caprine farming, and stud farming, as well as no increased risk for unspecified large animal farming. There were eight activities that exhibited sex differences, with risks higher for females than for males, with the exception of salt works/salt evaporation pond, and unspecified small animal farming.

## Discussion

In this study pertaining to the entire French FM population, certain agricultural activities were found to be more strongly associated with depression than other activities, overall and by sex category. The incidence of depression was found to be two times higher in females than males, as commonly reported in the literature.^21^ In our study, there were 28.2 depression cases per 1000 person-years, which is higher than in other studies and countries. Indeed, for depressive disorders, between 7.12 and 13.9 cases per 1000 person-years were found in the Spanish and UK general populations,^21^^,^^22^ while 23 cases per 1000 person-years were reported in a nationwide Danish work cohort.^23^ For major depression, between 2.3 and 15.9 cases per 1000 person-years were reported in the Swedish, Canadian, and US general populations.^24^

### Risk factors

The positive and negative associations that we found are likely to involve many risk factors, and more than one mechanism. Agricultural workers are affected in their daily life by different factors and stressors that could be harmful to their mental health, including but not limited to: isolation,^3^^,^^12^ health care accessibility and affordability,^3^^,^^4^^,^^12^^,^^25^ pesticide exposure,^3^^,^^9^^,^^13^^,^^17^^,^^26^ stress,^3^^,^^13^ quality and safety of food products,^12^ and Farmer's syndrome.^9^ FMs living together as a couple had higher risk of depression than single FMs (Table 2). Role conflict between farm and family (overlap between work and family environments) could potentially play a role in this observation. Indeed, difficulty to separate the private and professional life (poor work-life balance), with sometimes conflicting demands of work and family, the limited possibilities for relaxation and holidays, the fact that home and work are at the same location, and poor-quality relationship are associated with stress and depressive disorders in farmers.^3^^,^^4^^,^^12^ FMs with at least one employee had higher risk of depression than those with no employees. Several risk factors could potentially play a role in this observation. FMs with employees can experience interpersonal conflict, poor cooperation, poor workplace relationships with their employees, and can be subjected to higher financial stress (e.g., entitlement to pay their employees) than FMs with no employees, which were shown to be risk factors for depression.^3^^,^^4^^,^^12^^,^^27^

### Depression risks associated with agricultural activities

Increases in the risk of depression were observed in relation to several occupational agricultural activities performed by French FMs, suggesting that part of the risk could be attributable to occupational agricultural activities. Several studies have examined agricultural exposure and the risk of depression, by comparing most of the time overall agriculture with the general population.^1^ Only a few studies have investigated specific agricultural activities, mainly in the US, Australia and UK,^1^^,^^3^^,^^4^ using questionnaire data, and focusing on one specific occupation, such as dairy farming,^1^^,^^2^^,^^13^ or on the relation between pesticide exposures and the risk of depression.^11^^,^^12^^,^^17^^,^^26^ In France, data are scare, with only four studies that relied mainly on questionnaire data, pertain to a limited geographical area, and to less than 2200 individuals.^14^, ^15^, ^16^, ^17^

#### Pesticide use

Regarding the association between depression and pesticide use, several studies, with up to 21,208 pesticide applicators, found increased risks of depression, in particular among farmers applying herbicides,^17^^,^^26^^,^^28^^,^^29^ organophosphate and organochlorine insecticides,^3^^,^^26^^,^^28^, ^29^, ^30^ or fumigants.^26^^,^^28^^,^^29^ Most studies are from the Agricultural Health Study in the US, where a history of pesticide poisoning as well as high cumulative exposure to pesticides were associated with depression.^1^^,^^13^^,^^26^^,^^28^^,^^29^ We found a 10% excess risk of depression for male winegrowers, but not in other activities involving a frequent or high pesticide use such as arboriculture, or crop farming. We did not study depression risks related to specific pesticide compounds since these data were not collected by MSA. Regarding viticulture, besides potential pesticide exposure, other risk factors and stressors could play a role in the positive associations observed, such as weather conditions (e.g., drought), quality and safety of food products, time pressure, or plant and fruit diseases (e.g., mildew).^3^^,^^13^

#### Animal husbandry

The highest depression risks were found in our work for animal farming, with the exception of activities involving horses. A Norwegian study reported that male animal producers (n = 82) had the highest depression level among several farmer groups (n = 917 farmers).^31^ Most available data pertaining to animal farmers relate to dairy farmers.^2^^,^^11^^,^^12^^,^^14^^,^^32^ These studies, which includes between 30 and 985 dairy farmers, reported increased risks of depression in dairy farmers, as we observed. Many factors were proposed to explain the increased risks, such as disease outbreaks, economic crisis, taxes related to dairy production, quality and safety of food products, time pressure, and limited possibilities for relaxation and holidays.^12^ For instance, a study reported that among 661 Dutch dairy farmers experiencing animal culling from disease crisis, half of them suffered from severe post-traumatic distress.^25^ In our study, we found an increased risk in male cow famers compared to other agricultural activities. Cow farmers shared some risk factors with dairy farmers. Only one UK study examined the risk of depression in cow farmers, and in particular the impact of the beef crisis of 1996.^33^ This case–control study, conducted in 1996 several months after the beef crisis, reported that the crisis had no effect on the risk of depression of cattle farmers (n = 106), but concluded that a longer period may be required to assess a potential impact on mental health. Other animal farming, in particular pig, poultry and rabbit farming shared some of the aforementioned risk factors, which could potentially explained the increased risks we observed. One Swedish study reported increased risk of depression for pig farmers (n = 30),^11^ but we found no study reported an increased risk for poultry and rabbit farmers.

#### Other activities

Gardening, landscaping and reforestation companies are intensive jobs that involve a high degree of risk, in particular in chainsaw and skidder operators, climbing on trees, which was shown to lead to symptoms like anxiety, nervousness and lack of sleep,^34^ and could potentially play a role in the increased risk of depression we found.

### Sex differences

Sex differences were found for several agricultural activities (Table S3), suggesting that differences between females and males regarding occupational exposure, working tasks, behaviors, and risk factors existed, as already pointed out by several studies.^3^^,^^13^^,^^35^^,^^36^ A Japanese study including 273 dairy farmers showed that depression was associated with different occupational factors depending on sex.^35^ For instance, they reported that worries about one's financial situation, future of own farm, health status of livestock, and negative effects of work overload on own health were associated with depression in female. By contrast, concerns about the harmful effects of pesticides on health, and the balancing of family roles and work roles were related to depression in male. In our work, while most depression risks were higher in females than males, a few associations were stronger in males than females, which is unusual for depression.^3^

### Strengths and limitations of this work

Strengths of the study include a large nationwide population-based cohort from administrative data, with many exposed cases and completeness of available data (more than one million farmers for this study *vs.* <10,000 in the literature). While administrative data are increasingly used to conduct research on depression and inform health services and health policy,^7^ to the authors’ knowledge, this is the first study using data from insurance health databases pertaining to the entire French FM population.

Only persons under medical care can be identified with administrative health data, but less than one-third of depression diagnosis are recorded by physicians,^6^ which likely reduce the identification of depression in health records. We identified depression cases using ICD-10 codes and ATD prescriptions, which is not comparable to the real illness incidences, and could misestimate risk estimation. A review suggested that the best algorithm consists in including both ICD codes, and ATD prescriptions.^10^ However, regarding ICD-10 codes, their accuracy can vary depending on coding practices.^7^ Even though ATDs are predominantly used for the treatment of depressive disorders, they can be used to treat other diseases, including anxiety disorders, insomnia, and pain.^19^ ATD prescription also depends on the severity and form of depression, and is not necessarily the first line treatment, especially for mild forms of depression.^19^ Indications for the ATD prescriptions were not available. Therefore, data illustrates ATD prescriptions in a collective of insured patients, but it does not necessarily relate to ATD prescriptions in diagnosed patients with depression. Most ATDs prescribed were SSRIs, which is the most used ATD class for mental health problems.^19^ The sensitivity analysis restricted to SSRIs yielded similar results than the main analysis, which strengthen the assumption that only mental health problems were included. Because pre-existing medical comorbidities could be on the causal pathway from exposure to outcome and cause bias in adjustments, a sensitivity analysis excluding these confounders was performed. This sensitivity analysis yielded similar results than the main analysis, which indicates that adjusting on pre-existing medical comorbidities did not introduce bias.

The limited range of confounders available, which is often the case in administrative data,^18^ is one of the major limitations from this work. Confounding factors not available to TRACTOR could represent a bias, but their potential impact on the results is hard to evaluate, as these variables were not available. It is possible that their absence could bias the estimated effects, and confounds/masks the genuine relationship between agricultural activities and depression. In addition, regarding available confounders, their accuracy was sometimes limited, so the possibility of residual confounding cannot be excluded. Findings should therefore be considered carefully. Only an indirect exposure estimation was possible using activities from administrative databases, with no information on chemical, physical, or biological agents that could be encountered/used by FMs. Some activities were not descriptive enough to provide the best risk estimation possible, in particular for activities that are highly heterogeneous in nature, such as crop farming. Linking MSA data to other external sources (e.g., cohort studies, crop exposure matrices) could help to address some of the aforementioned limitations. A perspective from this work would be to use job and/or crop exposure matrices (JEMs) to try to ascertain more accurately pesticide exposure, physical, and/or psychosocial factors. However, this would be a challenging endeavor, with several potential bias, and limitations that would have to be addressed. For instance, the most relevant JEM(s) would have to be identified, which is not simple since there is no gold standard, and because the performance of a JEM depends on the exposure, and effect of interest.^37^, ^38^, ^39^ There will also be transcoding difficulties because the coding system of agricultural activities from MSA is not based on a standardized coding system, so the compatibility with existing JEMs might be limited. A more detailed discussion regarding the limitations of TRACTOR is described elsewhere.^18^

While this work allows for the identification of activities with a higher risk for depression than other type of farm work, it does not enable us to identify which factors contribute to these associations. To complement and go beyond this work, an additional study focusing on the identification of common/shared and differing factors determining depression for each agricultural activity is required, and will be conducted in the future. For instance, machine learning could be used to identify predictors of depression for each activity. Mixed-methods studies could also be conducted for agricultural activities that were found with the highest depression risks.

Because agricultural practices and risk factors can differ between countries, findings from this works might be unlikely to generalize beyond France. In addition, data were not available after 2016, therefore, it would be interesting to study how depression risks have evolved since then. Yet, we think our results are still relevant today as there is no other study reporting depression risks for specific agricultural activities, and because many of the aforementioned risk factors still remain topical.

Because we had only access to farmers, and not the whole population-at-risk, it was not possible to calculate the exact incidence rate of depression. However, our findings can help in identifying activities the most at risks of depression among the farming populations, where preventive measures could be potentially allocated in priority. A complementary perspective from this work could be to conduct the same work as we did, but by determining whether FMs have a higher depression risk compared to non-farming individuals.

### Public health implications

Large-scale efforts to identify depression and monitor its outcomes are of paramount importance to public health. Findings from this study will be of interest to policy makers and public health practitioners. Indeed, our findings could help to determine agricultural activities for which further studies (e.g., qualitative, quantitative, or mixed-methods) are needed in priority to identify depression risk factors, and appropriate preventive measures. Variability in the types of agricultural activities performed, implies that a comprehensive understanding of the topic is required to recommend relevant interventions. Identifying agricultural activities at risk of depression is thus a critical first step on the road to develop effective preventive measures against depression, but also injuries and suicide. Indeed, depression can inhibit safety behaviors leading to injuries,^13^ and can increase the risk for suicide.^9^^,^^17^^,^^29^ While prevention of depression is crucial, it requires effective interventions.^4^^,^^5^ There is a broad range of strategies for depression that exists, including evidence-based interventions such as therapeutic, psychological, and social interventions.^4^^,^^21^^,^^40^ Interventions combining more than one approaches were shown to be more efficient in reducing depression levels among employees in the workplace.^4^^,^^40^ Mental health literacy programs is an example of holistic/multi-component interventions that could be undertaken to improve knowledge, attitudes, and helping behaviors of FMs.^4^ The promotion of the creation of agricultural cooperatives is an example of targeted intervention that could be implemented in dairy farmers in order to secure markets, access supplies and services. To inform health services and health policy, the findings from this study will be presented nationally to the MSA, and to the Ministries of Agriculture and Food Sovereignty, Work, and Health and Solidarity. The present study was also conducted for each local MSA office (n = 35), with results that will be presented to all the local actors involved in mental health promotion and prevention. These presentations will allow us to exchange and confront our findings with the perceptions, and experiences of local actors, and discuss potential implications. After these exchanges, each MSA office will be able to determine whether additional resources should be allocated to screen for depression, to identify risk factors, and to implement tailored prevention measures and interventions for vulnerable groups at a local geographic scale.

### Conclusion

In conclusion, TRACTOR brings new insights and a wealth of information on the association of a wide range of agricultural activities and depression in FMs. This study suggests that certain agricultural activities are more strongly associated with depression than other activities, with differences between sexes. Our findings could act as a starting point for identifying potential targets for prevention and intervention. Further research regarding specific farming activities by sex category are however needed to confirm, understand, and identify which factors contribute to the observed risks in order to determine whether additional resources should be allocated and where, and which preventive measures should be implemented.

## Contributors

All authors meet all four criteria for authorship in the ICMJE Recommendations. The work reported in the paper has been performed by the authors, unless clearly specified in the text. All authors had full and direct access to all the data in the study, verified the underlying data in the study, and had final responsibility for the decision to submit for publication. All authors contributed to interpretation, reviewing, editing of the manuscript, and approved the submitted version. All authors affirms that the manuscript is an honest, accurate, and transparent account of the study being reported; that no important aspects of the study have been omitted; and that any potential discrepancies from the study as originally planned (and, if relevant, registered) have been explained.

**Pascal Petit**: Conceptualization, Methodology, Software, Validation, Formal analysis, Investigation, Data Curation, Writing - Original Draft, Writing - Review & Editing, Visualization, Supervision, Funding acquisition, Project administration.

**Gérald Gandon**: Methodology, Validation, Writing - Review & Editing.

**Marc Dubuc**: Methodology, Writing - Review & Editing.

**Nicolas Vuillerme**: Methodology, Writing - Review & Editing.

**Vincent Bonneterre**: Methodology, Validation, Writing - Review & Editing, Supervision, Funding acquisition, Project administration.

## Data sharing statement

The data (participant data, data dictionary, other related document) that support the findings of this study are not publicly available. Reasonable request to the Mutualité Sociale Agricole (MSA) can be made but restrictions applied to the availability of these data due to the individual and medical nature of the data, which requires the approval from both the MSA and the French independent administrative authority protecting privacy and personal data (CNIL). Further information is available from the corresponding author upon request. The data used for this article and for the TRACTOR project were provided by the MSA following standard secure regulatory procedures. The use of MSA data for the TRACTOR project was approved by the French independent administrative authority protecting privacy and personal data (CNIL) (authorization number MMS/SBM/AE171001). Patient consent was not required as the research team accessed fully anonymized data only, which were collected as part of routine health insurance purpose activities by the MSA. In addition, results were reported at a large collective scale (i.e., farming activity level) and measures were undertaken to prevent the risk of reidentification of individuals. Following CNIL instructions, MSA is required to make bill posting in each of its 35 offices and to communicate yearly to all of its insured individuals about the goals, advancements and achievements of the TRACTOR project.

## Declaration of interests

The authors declare that they have no known competing financial interests or personal relationships that could have appeared to influence the work reported in this paper. The paper contents, including any opinions and/or conclusions expressed, are solely those of the authors.

## Appendix A Supplementary data

Supplementary Fig. S5

Translated abstract

## References

[1] Hagen B.N.M., Albright A., Sargeant J. (2019). Research trends in farmers' mental health: a scoping review of mental health outcomes and interventions among farming populations worldwide. PLoS One.

[2] Onwuameze O.E., Paradiso S., Peek-Asa C., Donham K.J., Rautiainen R.H. (2013). Modifiable risk factors for depressed mood among farmers. Ann Clin Psychiatr.

[3] Yazd S.D., Wheeler S.A., Zuo A. (2019). Key risk factors affecting farmers' mental health: a systematic review. Int J Environ Res Publ Health.

[4] Younker T., Radunovich H.L. (2021). Farmer mental health interventions: a systematic review. Int J Environ Res Publ Health.

[5] Pearce M., Garcia L., Abbas A. (2022). Association between physical activity and risk of depression A systematic review and meta-analysis. JAMA Psychiatr.

[6] Doktorchik C., Patten S., Eastwood C. (2019). Validation of a case definition for depression in administrative data against primary chart data as a reference standard. BMC Psychiatr.

[7] Fiest K.M., Jette N., Quan H. (2014). Systematic review and assessment of validated case definitions for depression in administrative data. BMC Psychiatr.

[8] Hagen B.N.M., Winder C.B., Wootten J., McMullen C.K., Jones-Bitton A. (2020). A systematic review and meta-analysis of depression among farming populations worldwide. Int J Environ Res Publ Health.

[9] Joo Y., Roh S. (2016). Risk factors associated with depression and suicidal ideation in a rural population. Environ Health Toxicol.

[10] Townsend L., Walkup J.T., Crystal S., Olfson M. (2012). A systematic review of validated methods for identifying depression using administrative data. Pharmacoepidemiol Drug Saf.

[11] Kolstrup C., Lundqvist P., Pinzke S. (2008). Psychosocial work environment among employed Swedish dairy and pig farmworkers. J Agromed.

[12] Kolstrup C.L., Kallioniemi M., Lundqvist P., Kymalainen H.R., Stallones L., Brumby S. (2013). International perspectives on psychosocial working conditions, mental health, and stress of dairy farm operators. J Agromed.

[13] Reed D.B., Claunch D.T. (2020). Risk for depressive symptoms and suicide among U.S. Primary farmers and family members: a systematic literature review. Workplace Health Saf.

[14] Guillien A., Laurent L., Soumagne T. (2017). Anxiety and depression among dairy farmers: the impact of COPD. Int J Chron Obstruct Pulmon Dis.

[15] Khireddine-Medouni I., Rabet G., Deschamps G., Geoffroy-Perez B. (2019). Prévalence de la symptomatologie dépressive et exposition aux facteurs professionnels psychosociaux chez les actifs affiliés à la Mutualité sociale agricole de cinq départements en 2010 : résultats de la phase pilote de la cohorte Coset-MSA. Bull Epidemiol Hebd.

[16] Pérès K., Matharan F., Allard M. (2012). Health and aging in elderly farmers: the AMI cohort. BMC Publ Health.

[17] Weisskopf M.G., Moisan F., Tzourio C., Rathouz P.J., Elbaz A. (2013). Pesticide exposure and depression among agricultural workers in France. Am J Epidemiol.

[18] Petit P., Bosson-Rieutort D., Maugard C. (2022). The TRACTOR project: TRACking and MoniToring occupational risks in agriculture using French insurance health data (MSA). Ann Work Expo Health.

[19] Haller E., Watzke B., Blozik E. (2019). Antidepressant prescription practice and related factors in Switzerland: a cross-sectional analysis of health claims data. BMC Psychiatr.

[20] Petit P., Bicout D.J. (2022). Health risk assessment with multiple reference indexes. Sci Total Environ.

[21] Martin-Merino E., Ruigomez A., Johansson S., Wallander M.A., Garcia-Rodriguez L.A. (2010). Study of a cohort of patients newly diagnosed with depression in general practice: prevalence, incidence, comorbidity, and treatment patterns. Prim Care Companion J Clin Psychiatry.

[22] Vieta E., Alonso J., Perez-Sola V. (2021). Epidemiology and costs of depressive disorder in Spain: the EPICO study. Eur Neuropsychopharmacol.

[23] Madsen I.E.H., Svane-Petersen A.C., Holm A. (2021). Work-related violence and depressive disorder among 955,573 employees followed for 6.99 million person-years. The Danish Work Life Course Cohort study: work-related violence and depression. J Affect Disord.

[24] Yildirim M., Gaynes B.N., Keskinocak P., Pence B.W., Swann J. (2022). DIP: natural history model for major depression with incidence and prevalence. J Affect Disord.

[25] Patel V., Chisholm D., Parikh R. (2016). Addressing the burden of mental, neurological, and substance use disorders: key messages from Disease Control Priorities, 3rd edition. Lancet.

[26] Beard J.D., Umbach D.M., Hoppin J.A. (2014). Pesticide exposure and depression among male private pesticide applicators in the agricultural health study. Environ Health Perspect.

[27] Battams S., Roche A.M., Fischer J.A., Lee N.K., Cameron J., Kostadinov V. (2014). Workplace risk factors for anxiety and depression in male-dominated industries: a systematic review. Health Psychol Behav Med.

[28] Cancino J., Soto K., Tapia J. (2023). Occupational exposure to pesticides and symptoms of depression in agricultural workers. A systematic review. Environ Res.

[29] Zanchi M.M., Marins K., Zamoner A. (2023). Could pesticide exposure be implicated in the high incidence rates of depression, anxiety and suicide in farmers? A systematic review. Environ Pollut.

[30] Wu Y., Song J., Zhang Q. (2022). Association between organophosphorus pesticide exposure and depression risk in adults: a cross-sectional study with NHANES data. Environ Pollut.

[31] Sanne B., Mykletun A., Moen B.E., Dahl A.A., Tell G.S. (2004). Farmers are at risk for anxiety and depression: the Hordaland Health Study. Occup Med (Lond).

[32] Olff M., Koeter M.W.J., Heleen Van Haaften E., Kersten P.H., Gersons B.P.R. (2005). Impact of a foot and mouth disease crisis on post-traumatic stress symptoms in farmers. Br J Psychiatry.

[33] Eisner C.S., Neal R.D., Scaife B. (1999). The effect of the 1996 ‘beef crisis’ on depression and anxiety in farmers and non-farming controls. Br J Gen Pract.

[34] Lotfalian M., Emadian S.F., Far Riahi N., Salimi M., Sheikhmoonesi F. (2012). Occupational stress impact on mental health status of forest workers. Middle East J Sci Res.

[35] Sato M., Kato H., Noguchi M., Ono H., Kobayashi K. (2020). Gender differences in depressive symptoms and work environment factors among dairy farmers in Japan. Int J Environ Res Publ Health.

[36] Viertio S., Kiviruusu O., Piirtola M. (2021). Factors contributing to psychological distress in the working population, with a special reference to gender difference. BMC Publ Health.

[37] Burstyn I., Lavoue J., Van Tongeren M. (2012). Aggregation of exposure level and probability into a single metric in job-exposure matrices creates bias. Ann Occup Hyg.

[38] Peters S. (2020). Although a valuable method in occupational epidemiology, job-exposure -matrices are no magic fix. Scand J Work Environ Health.

[39] Fadel M., Evanoff B.A., Andersen J.H. (2020). Not just a research method: if used with caution, can job-exposure matrices be a useful tool in the practice of occupational medicine and public health?. Scand J Work Environ Health.

[40] Wan Mohd Yunus W.M.A., Musiat P., Brown J.S.L. (2018). Systematic review of universal and targeted workplace interventions for depression. Occup Environ Med.

