# Supplementary material for: Agricultural activities and risk of treatment for depressive disorders among the entire French agricultural workforce: the TRACTOR project, a nationwide retrospective cohort study
Source: Lancet Reg Health Eur. 2023 Jun 26;31:100674. doi: 10.1016/j.lanepe.2023.100674 (PMC10318497; doi:10.1016/j.lanepe.2023.100674)
Supplement: Supplementary material [file mmc1.pdf]

Supplemental material

Title
Agricultural activities and risk of treatment for depression depressive disorders among the entire French agricultural workforce: the TRACTOR project, a nationwide retrospective cohort study

Authors
Pascal Petit, Gérald Gandon, Marc Dubuc, Nicolas Vuillerme, Vincent Bonneterre

Table of contents

Fig. S1: Decision rule to consider depression as potentially work-related 2

Fig.S2: Pareto chart of the number of farm managers per number of ATD prescriptions 3

Fig. S3: Number of farm managers with a depression identified with LTI declaration or antidepressant prescription 4

Fig. S4: Number of farm managers prescribed with antidepressants per agent 5

Fig. S5: Number of antidepressant agents prescribed to farm managers 6

Table S1: List and definition of agricultural activities available using MSA databases 7

Table S2: Agricultural activities and risks of depression, TRACTOR, France, 2002-2016 – Results for each algorithm for the main analysis 8

Table S3: Results of the interaction tests regarding the sex 8

Table S4: Agricultural activities and risks of depression, TRACTOR, France, 2002-2016 - results from the sensitivity analysis 1 9

Table S5: Agricultural activities and risks of depression, TRACTOR, France, 2002-2016 - results from the sensitivity analysis 2 10

Table S6: Agricultural activities and risks of depression, TRACTOR, France, 2002-2016 - results from the sensitivity analysis 3 11

Table S7: Agricultural activities and risks of depression, TRACTOR, France, 2002-2016 - results from the sensitivity analysis 4 restricted to SSRIs 12

STROBE Statement Checklist 13

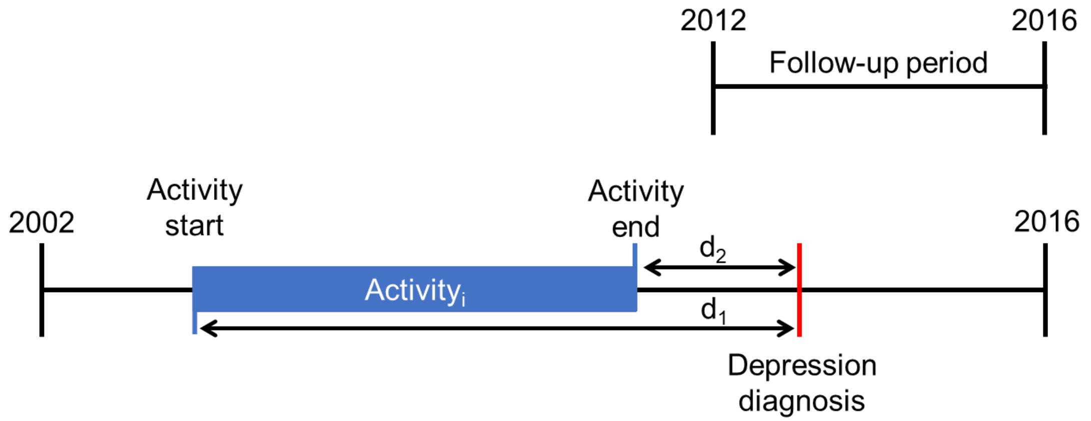

Depression considered as work-related if:

- $d_1 > 0$ year and $d_2 \leq 2$ years → for the main analysis, and sensitivity analyses 1, 3 and 4
- $d_1 > 0$ year → for sensitivity analysis 2

Fig. S1: Decision rule to consider depression as potentially work-related

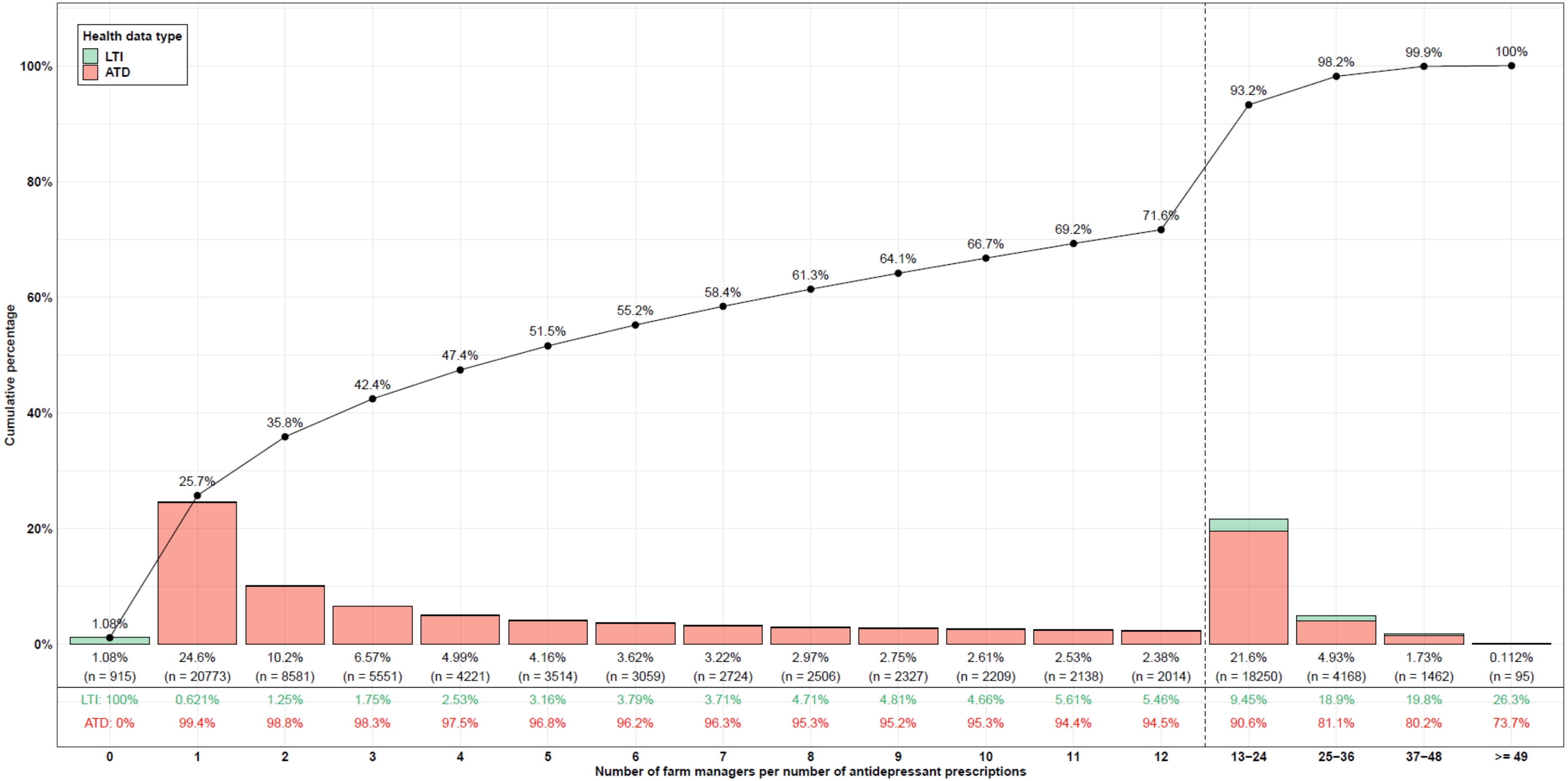

Fig.S2: Pareto chart of the number of farm managers per number of ATD prescriptions

ATD: antidepressant, LTI: long-term illness.

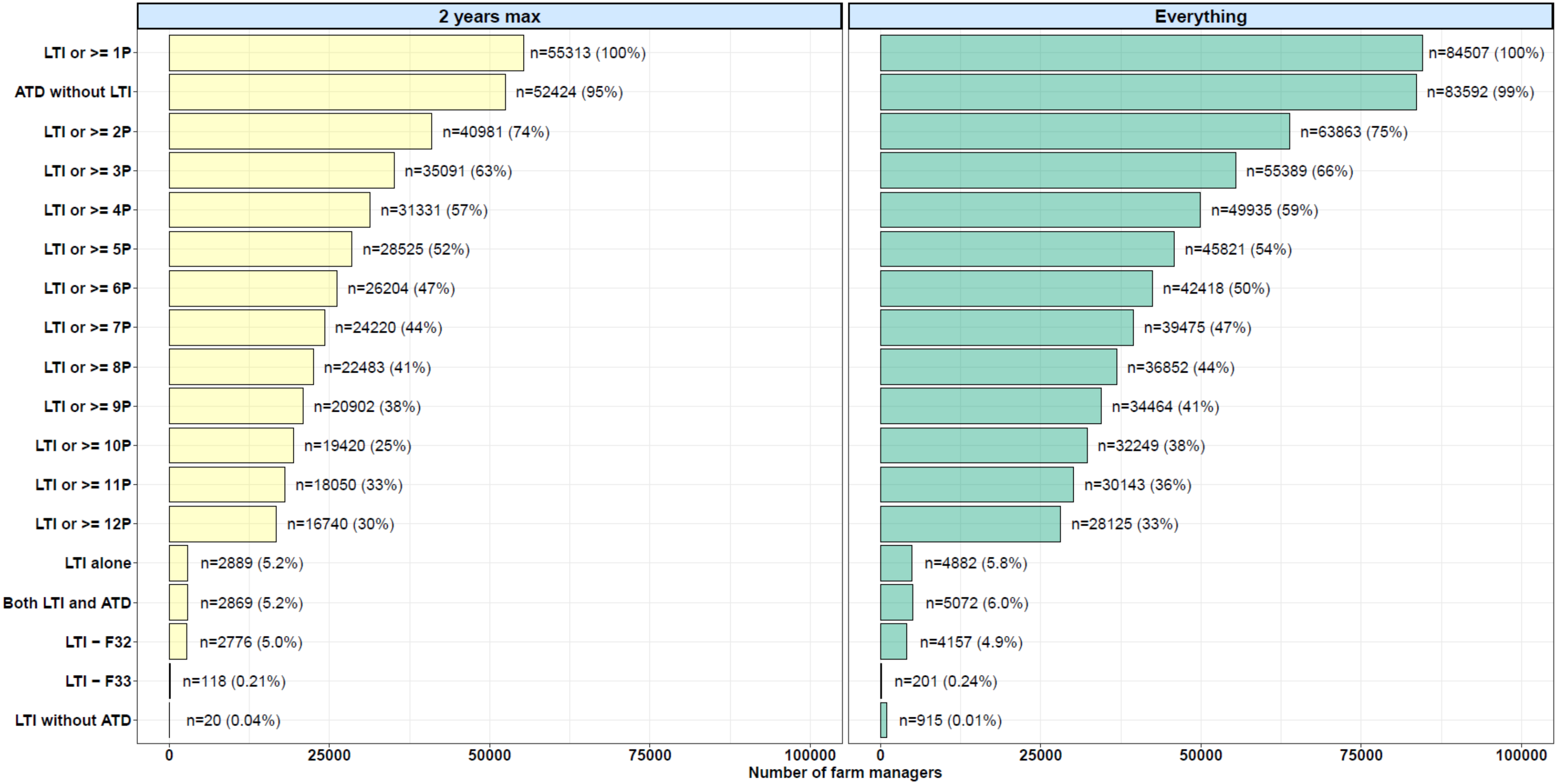

Fig. S3: Number of farm managers with a depression identified with LTI declaration or antidepressant prescription

ATD: antidepressant, F32: insurance declaration for depressive episode, F33: insurance declaration for major depressive disorder, recurrent, LTI: long term illness insurance declaration for depression, P: antidepressant prescription.

2 years max: The yellow bars refer to farm managers that were considered as depression cases for the main analysis, for which a restriction of time was applied to consider depression as work-related. Indeed; Only farm managers that had a depression insurance declaration or antidepressant prescription claim after the start of their activity, but no more than two years after the end of their activity were considered as depression cases.

Everything: The green bars refer to farm managers that were considered as depression for the sensitivity analysis, for which no restriction of time was applied to consider depression as work-related.

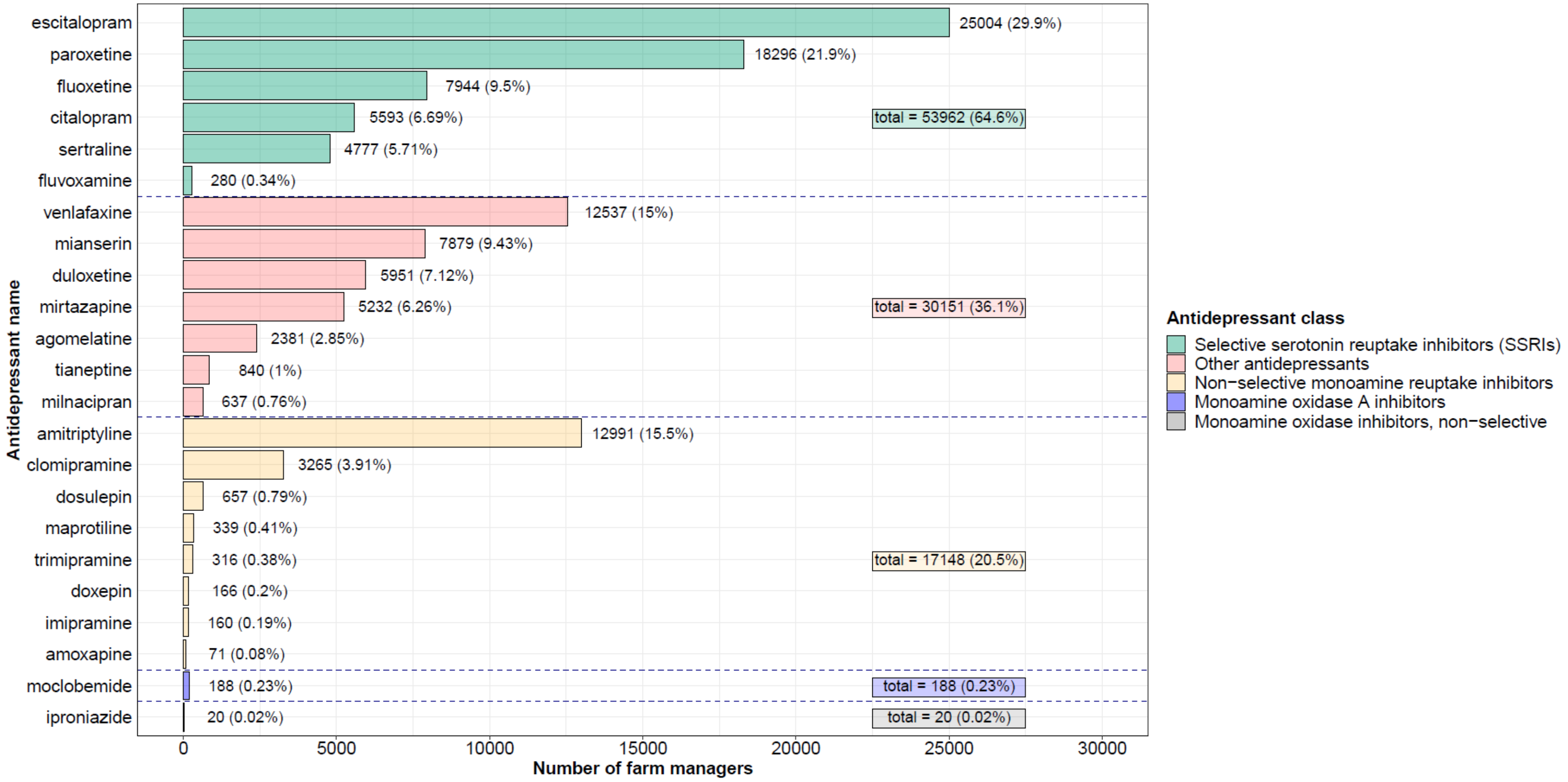

Fig. S4: Number of farm managers prescribed with antidepressants per agent

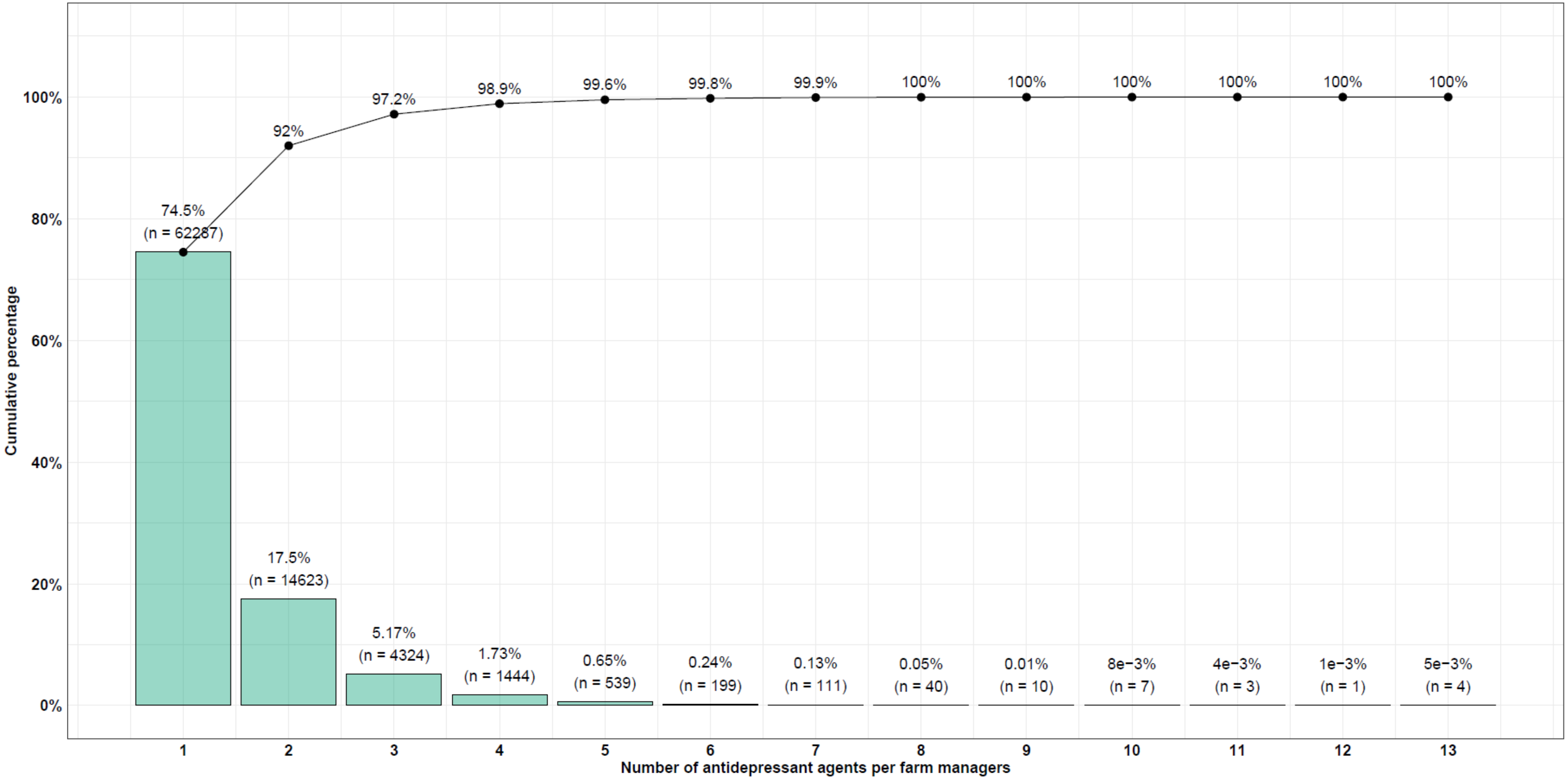

Fig. S5: Number of antidepressant agents prescribed to farm managers

Table S1: List and definition of agricultural activities available using MSA databases

Activity name	Definition	Example of task
Truck farming, floriculture/flower-growing	Practice of growing and harvesting fruits, vegetables, and flowers for sale	Proper crop selection, soil and water management, pest and disease management, harvesting and post-harvest handling
Fruit arboriculture	Cultivation of fruits growing on trees	Pruning branches, removing damaged or diseased branches, fertilizing trees, monitoring and controlling pests and diseases
Garden center/tree nursery	Propagation, breeding and growing of plants and trees to a desired size	Planting, watering, weeding, digging, using tools such as sieve, or spade
Crop farming (e.g., wheat, corn, and industrial grower)	Cultivation of plants (e.g. cereals, vegetables) for food	Monitoring and controlling pests and diseases, fertilizing, harvesting and post-harvest handling, grading, soil and water management, tractor driving
Viticulture	Cultivation and harvesting of grapes, and winemaking	Monitoring and controlling pests and diseases, fertilizing, soil and water management, canopy management, monitoring fruit development and characteristics, harvesting and post-harvest handling, vine pruning
Sylviculture/forestry (e.g., thinning, pruning)	Controlling the growth, composition/structure, as well as quality of forests	Thinning, pruning, spacing
Unspecified specialized farming (e.g., herbs, mushrooms)	Cultivation and harvesting of herbs, spices, medical plants, and mushrooms	Layering, cutting, grafting, pruning, mulching, weed management
Dairy farming	Raising and breeding of domestic cows for milk production	Breeding, milking (manually, or using machine), feeding, bulk handling, disease prevention, managing waste
Cow farming	Raising and breeding of domestic cows as livestock	Feeding, maintaining facilities, monitoring the herd for signs of illness, assisting with calving, performing artificial insemination, and managing waste
Both/mixed dairy and cow farming	Raising and breeding of domestic cows as livestock and for milk production	Breeding, milking, feeding, bulk handling, disease prevention
Ovine and caprine farming	Raising and breeding of domestic sheep and goats	Breeding, milking, wool production, maintenance of the property, mustering, milking, shearing and drenching livestock, sterilizing machines, and collecting, grading and packaging produce
Pig farming	Raising and breeding of domestic pigs as livestock	Maintain equipment and buildings, spread manure, feeding, monitoring the pigs for signs of illness, assist in artificial insemination, transporting stock to farms or processing plants
Stud farming	Raising and breeding of domestic horses	Feeding, breeding, assist in artificial insemination, disease prevention
Unspecified large animal farming (e.g., ostrich, llama)	Raising and breeding of large animals such as wild animals, ostrich, bison, boar, or llama	Feeding, breeding, disease prevention
Poultry and rabbit farming	Raising and breeding of domestic birds and rabbits	Feeding, breeding, disease prevention, assist in artificial insemination, gathering eggs, cleaning rooms and equipment/facility maintenance
Unspecified small animal farming (e.g., frogs, snails, bees)	Raising and breeding of small animals such as frogs, bees, insects, minks, beavers, or snails	Feeding, breeding, disease prevention
Training, dressage, riding clubs	Activity including training and dressage of domestic equines	Feeding, disease prevention, teaching, maintenance of the property, tourism
Shellfish farming (e.g., oyster farming, scallop aquaculture)	Cultivation and harvest of aquatic invertebrates, such as oysters, clams and mussels	Breeding, rearing, harvesting, using a rack, processing, and shipment
Unspecified and mixed farming (e.g., polyculture, mixed farming, diversified farming)	Activity involving both the growing of crops and the raising of livestock, or the growing of more than one crop species	Planning and coordinating the production and marketing of crops and livestock, breeding and raising livestock for the production of meat, wool, skins, milk or eggs, and growing grain, seed crops, vegetables, fruit or nuts
Salt works/salt evaporation pond	Production of salt from sea water	Harvesting, filtering
Wood production (e.g., lopping)	Harvesting of round wood (trunk and branch wood) from coniferous (softwood) and non-coniferous (hardwood) trees	Harvesting, lopping, transport
Stationary sawmill (e.g., edging, trimming, decking, debarking)	Cutting logs into lumber using large bandsaw blades	Edging, trimming, decking, debarking, use of machines
Agricultural work companies (e.g., pesticide applications, harvest reaping)	Companies that assist or perform farm works such as harvest reaping	Harvest reaping, harrowing, sowing seeds, pesticide application, tilling, clearing
Gardening, landscaping and reforestation companies	Companies that perform gardening, landscaping and reforestation works	Gardening, landscaping, chainsaw and skidder operation, climbing on trees, monitoring and controlling pests and diseases
Company representative/authorized representative	Self-employed insurance broker responsible for representing a legal entity (agricultural insurance company)	Traveling, administrative tasks, visiting farms
Rural craftsperson (e.g., mason, mechanics)	Self-employed person performing construction or hand-crafted works in rural areas, including jobs such as well-diggers, painters, electricians, masons, carpenters, saddlers, blacksmiths, mechanics, wheelwright or farriers	Fixing agricultural machinery, painting, welding, hammering

Table S2: Agricultural activities and risks of depression, TRACTOR, France, 2002-2016 – Results for each algorithm for the main analysis

Please refer to the SM Excel file entitled Table S2.

Table S3: Results of the interaction tests regarding the sex

**Activity**	**Main analysis**		**SA 1**		**SA 2**		**SA 3**		**SA 4**	
	**d**	**p**	**d**	**p**	**d**	**p**	**d**	**p**	**d**	**p**
Truck farming, floriculture/flower-growing	F > M	0·09	F > M	0·07	M > F	0·11	F > M	**< 0·0001**	F > M	0·16
Fruit arboriculture	M > F	0·17	M > F	0·09	F > M	**< 0·0001**	M > F	0·12	M > F	0·22
Garden center/tree nursery	M > F	0·32	F > M	0·37	F > M	**0·03**	F > M	0·40	F > M	0·26
Crop farming (e.g., wheat, corn, and industrial grower)	M > F	**0·04**	M > F	**1·5e-3**	M > F	**< 0·0001**	M > F	**1·2e-3**	M > F	0·13
Viticulture	M > F	0·67	M > F	0·21	M > F	**< 0·0001**	M > F	0·19	M > F	0·40
Sylviculture/forestry (e.g., thinning, pruning)	M > F	0·89	M > F	0·78	F > M	**0·01**	M > F	0·60	F > M	0·34
Unspecified specialized farming (e.g., herbs, mushrooms)	M > F	0·67	M > F	0·76	M > F	**0·02**	M > F	0·72	M > F	0·47
Dairy farming	F > M	**9·4e-3**	F > M	**0·05**	F > M	**< 0·0001**	F > M	**5·5e-3**	F > M	**1·9e-3**
Cow farming	F > M	**< 0·0001**	F > M	**9·8e-4**	F > M	**5·9e-4**	F > M	**6·6e-4**	F > M	**0·01**
Both/mixed dairy and cow farming	F > M	**0·05**	F > M	0·11	M > F	0·60	F > M	**0·04**	F > M	0·23
Ovine and caprine farming	F > M	0·38	F > M	0·56	M > F	**2·0e-4**	F > M	0·70	M > F	0·48
Pig farming	F > M	**0·03**	F > M	**0·04**	M > F	0·12	F > M	**0·03**	F > M	0·28
Stud farming	M > F	0·39	M > F	0·38	M > F	0·55	M > F	0·71	M > F	0·72
Training, dressage, riding clubs	M > F	**0·03**	F > M	**0·02**	M > F	**0·04**	F > M	0·07	F > M	**0·05**
Unspecified large animal farming (e.g., ostrich, llama)	M > F	0·32	M > F	0·33	F > M	0·47	F > M	0·36	F > M	0·34
Poultry and rabbit farming	F > M	0·47	F > M	0·59	F > M	**4·5e-4**	F > M	0·56	F > M	0·23
Unspecified small animal farming (e.g., frogs, snails, bees)	M > F	**2·1e-3**	M > F	**1·6e-3**	F > M	**< 0·0001**	M > F	**9·0e-4**	M > F	**0·02**
Shellfish farming (e.g., oyster farming, scallop aquaculture)	F > M	0·78	M > F	0·83	F > M	0·10	M > F	0·53	M > F	0·81
Unspecified and mixed farming (e.g., polyculture, mixed farming, diversified farming)	M > F	**1·0e-4**	F > M	**2·2e-3**	F > M	**3·4e-3**	F > M	**3·0e-4**	F > M	**9·0e-3**
Salt works/salt evaporation pond	F > M	**0·02**	M > F	**0·02**	F > M	**< 0·0001**	M > F	**0·01**	M > F	**0·04**
Wood production (e.g., lopping)	F > M	**0·03**	F > M	**0·04**	F > M	0·49	F > M	**0·04**	F > M	0·10
Stationary sawmill (e.g., edging, trimming, decking, debarking)	F > M	0·08	F > M	0·08	F > M	**0·03**	F > M	0·08	F > M	**0·03**
Agricultural work companies (e.g., pesticide applications, harvest reaping)	F > M	**0·05**	F > M	0·06	M > F	0·61	F > M	**0·05**	M > F	0·46
Gardening, landscaping and reforestation companies	M > F	**3·2e-03**	F > M	**6·1e-3**	F > M	0·24	F > M	**0·01**	F > M	**5·4e-3**
Company representative/authorized representative	M > F	0·74	M > F	0·73	M > F	0·72	M > F	0·73	M > F	0·55
Rural craftsperson (e.g., mason, mechanics)	M > F	0·50	M > F	0·50	M > F	**0·02**	M > F	0·53	M > F	0·60

Note: d: difference direction, F > M: depression risk higher for females than males, M > F: depression risk higher for males than females, p: p-value, SA: sensitivity analysis. The main analysis adjusted for sex, age, first year of the farm's establishment, and the number of working years. The sensitivity analysis 1 included the same covariates as the main analysis, with the exception of the number of pre-existing comorbidities. The sensitivity analysis 2 adjusted on the same variables than the main analysis, but it was considered that depression cases could be work-related for all FMs that had a depression insurance declaration or ATD prescription claim, regardless of the date it occurred after the start of their activity (Fig. S1). The third sensitivity analysis adjusted on the same covariates than the main analysis in addition to other potential cofounders, that were selected based on the variance inflation factor (VIF) (VIF≤ 2·5) to remove collinear variables. The fourth sensitivity analysis was restricted to selective serotonin reuptake inhibitors (SSRIs) (Table 1).

Table S4: Agricultural activities and risks of depression, TRACTOR, France, 2002-2016 - results from the sensitivity analysis 1

**Activity**	**Both sexes** (n=1088561)					**Female** (n=337263)					**Male** (n=751298)				
	**n (%)**	**m (range)**	**HR**	**p**	**p.adj**	**n (%)**	**m (range)**	**HR**	**p**	**p.adj**	**n (%)**	**m (range)**	**HR**	**p**	**p.adj**
Truck farming, floriculture/flower-growing	43487 (3·99)	80; 1855	1·08 [1·00-1·16]	0·10	0·13	13251 (3·93)	26; 728	1·12 [1·01-1·24]	0·10	0·12	30236 (4·02)	54; 1127	1·05 [0·95-1·18]	0·40	0·56
Fruit arboriculture	25001 (2·3)	51; 1027	0·92 [0·83-1·02]	0·14	0·22	7927 (2·35)	21; 405	**0·81 [0·69-0·96]**	0·04	**0·05**	17074 (2·27)	30; 622	1·00 [0·88-1·14]	0·81	0·96
Garden center/tree nursery	5380 (0·49)	11; 282	1·23 [1·01-1·51]	0·11	0·13	1419 (0·42)	4; 95	1·27 [0·93-1·79]	0·20	0·25	3961 (0·53)	7; 187	1·20 [0·94-1·56]	0·20	0·27
Crop farming (e.g., wheat, corn, and industrial grower)	316926 (29·1)	547; 10755	**0·83 [0·80-0·85]**	< 0·0001	**< 0·0001**	105642 (31·3)	261; 4272	**0·80 [0·76-0·84]**	< 0·0001	**< 0·0001**	211284 (28·1)	286; 6483	**0·87 [0·84-0·91]**	< 0·0001	**< 0·0001**
Viticulture	121950 (11·2)	266; 6531	1·02 [0·98-1·06]	0·52	0·71	42909 (12·7)	112; 2825	0·99 [0·93-1·05]	0·50	0·74	79041 (10·5)	154; 3706	1·05 [1·00-1·11]	0·10	0·13
Sylviculture/forestry (e.g., thinning, pruning)	2160 (0·2)	6; 80	1·31 [0·93-1·89]	0·17	0·24	359 (0·11)	4; 19	1·26 [0·68-2·35]	0·48	0·75	1801 (0·24)	2; 61	1·49 [1·01-2·22]	0·08	0·11
Unspecified specialized farming (e.g., herbs, mushrooms)	6615 (0·61)	22; 271	**1·28 [1·07-1·53]**	9·9e-3	**0·01**	2408 (0·71)	11; 122	1·17 [0·90-1·52]	0·27	0·37	4207 (0·56)	11; 149	**1·33 [1·04-1·73]**	0·04	**0·05**
Dairy farming	161436 (14·8)	545; 9135	**1·30 [1·25-1·35]**	< 0·0001	**< 0·0001**	49659 (14·7)	228; 3615	**1·33 [1·26-1·41]**	< 0·0001	**< 0·0001**	111777 (14·9)	317; 5520	**1·20 [1·14-1·26]**	< 0·0001	**< 0·0001**
Cow farming	111873 (10·3)	481; 6933	**1·44 [1·38-1·49]**	< 0·0001	**< 0·0001**	32904 (9·76)	203; 2840	**1·55 [1·46-1·63]**	< 0·0001	**< 0·0001**	78969 (10·5)	278; 4093	**1·32 [1·26-1·39]**	< 0·0001	**< 0·0001**
Both/mixed dairy and cow farming	31070 (2·85)	98; 1834	**1·14 [1·06-1·23]**	0·01	**0·01**	8084 (2·4)	37; 646	**1·24 [1·10-1·40]**	0·02	**0·02**	22986 (3·06)	61; 1188	1·04 [0·95-1·15]	0·42	0·64
Ovine and caprine farming	48716 (4·48)	150; 2327	1·05 [0·99-1·12]	0·17	0·24	17314 (5·13)	70; 1093	1·06 [0·97-1·17]	0·31	0·42	31402 (4·18)	80; 1234	1·04 [0·96-1·14]	0·39	0·58
Pig farming	13636 (1·25)	42; 845	**1·21 [1·08-1·35]**	0·02	**0·02**	3890 (1·15)	20; 345	**1·42 [1·20-1·69]**	4·1e-3	**4·2e-3**	9746 (1·3)	22; 500	1·06 [0·92-1·22]	0·39	0·62
Stud farming	17164 (1·58)	33; 671	1·00 [0·87-1·15]	0·53	0·79	7408 (2·2)	20; 402	0·93 [0·78-1·12]	0·41	0·60	9756 (1·3)	13; 269	1·12 [0·91-1·39]	0·34	0·50
Unspecified large animal farming (e.g., ostrich, llama)	14874 (1·37)	19; 742	0·95 [0·81-1·11]	0·36	0·46	6619 (1·96)	9; 411	0·95 [0·79-1·16]	0·39	0·52	8255 (1·1)	10; 331	0·94 [0·75-1·20]	0·49	0·74
Poultry and rabbit farming	2997 (0·28)	5; 149	**1·83 [1·44-2·36]**	0·02	**0·02**	1437 (0·43)	3; 93	**1·64 [1·20-2·27]**	0·03	**0·03**	1560 (0·21)	2; 56	**1·85 [1·24-2·77]**	0·04	**0·04**
Unspecified small animal farming (e.g., frogs, snails, bees)	25540 (2·35)	81; 1620	**1·30 [1·20-1·42]**	1·3e-3	**1·4e-3**	9995 (2·96)	39; 824	**1·33 [1·19-1·50]**	0·01	**0·01**	15545 (2·07)	42; 796	**1·28 [1·14-1·45]**	5·4e-3	**5·9e-3**
Training, dressage, riding clubs	19896 (1·83)	31; 614	**0·81 [0·70-0·94]**	0·03	**0·04**	8596 (2·55)	10; 277	**0·55 [0·44-0·69]**	1·3e-3	**1·4e-3**	11300 (1·5)	21; 337	1·13 [0·95-1·35]	0·19	0·30
Shellfish farming (e.g., oyster farming, scallop aquaculture)	3795 (0·35)	7; 159	1·33 [1·02-1·74]	0·08	0·10	725 (0·22)	2; 45	1·25 [0·82-1·92]	0·34	0·52	3070 (0·41)	5; 114	**1·54 [1·13-2·14]**	0·03	**0·04**
Unspecified and mixed farming (e.g., polyculture, mixed farming, diversified farming)	127900 (11·7)	346; 6690	**1·21 [1·17-1·26]**	1·4e-3	**1·5e-3**	39338 (11·7)	144; 2679	**1·26 [1·19-1·34]**	4·2e-3	**4·3e-3**	88562 (11·8)	202; 4011	**1·13 [1·07-1·20]**	0·01	**0·01**
Salt works/salt evaporation pond	974 (0·09)	2; 40	0·89 [0·55-1·45]	0·65	0·90	221 (22·7)	0; 3	**0·36 [0·15-0·94]**	0·04	**0·04**	753 (0·1)	2; 34	1·35 [0·82-2·25]	0·25	0·38
Wood production (e.g., lopping)	11385 (1·05)	13; 504	1·08 [0·91-1·29]	0·37	0·50	297 (0·09)	0; 22	**1·99 [1·10-3·61]**	0·03	**0·05**	11088 (1·48)	13; 482	1·11 [0·93-1·34]	0·26	0·36
Stationary sawmill (e.g., edging, trimming, decking, debarking)	787 (0·07)	3; 45	1·52 [0·95-2·54]	0·18	0·23	52 (0·02)	0; 52	**3·13 [1·26-8·02]**	0·04	**0·05**	735 (0·1)	3; 37	1·37 [0·82-2·38]	0·33	0·45
Agricultural work companies (e.g., pesticide applications, harvest reaping)	15557 (1·43)	20; 656	1·12 [0·97-1·31]	0·18	0·23	1866 (0·55)	3; 107	1·33 [0·98-1·84]	0·09	0·12	13691 (1·82)	17; 549	1·11 [0·94-1·33]	0·29	0·36
Gardening, landscaping and reforestation companies	48878 (4·49)	76; 2309	**1·26 [1·15-1·38]**	0·02	**0·02**	2531 (0·75)	9; 169	**1·55 [1·19-2·04]**	0·02	**0·02**	46347 (6·17)	67; 2140	**1·43 [1·31-1·57]**	5·1e-3	**5·1e-3**
Company representative/authorized representative	1931 (0·18)	2; 42	0·85 [0·56-1·30]	0·46	0·70	1496 (0·44)	1; 35	0·80 [0·51-1·26]	0·34	0·53	437 (0·06)	1; 7	1·15 [0·41-3·32]	0·74	0·96
Rural craftsperson (e.g., mason, mechanics)	7701 (0·71)	0; 5	**0·03 [0·01-0·07]**	< 0·0001	**< 0·0001**	283 (0·08)	0	NC	NC	NC	7418 (0·99)	0; 5	**0·03 [0·01-0·08]**	< 0·0001	**< 0·0001**

Note: HR: hazard ratio, n: number of exposed farm managers, m: range of the number of exposed depression cases, NC: not calculated, p: p-value, p.adj: p-value adjusted using the Benjamini-Hochberg approach. Hazard ratios were estimated by Cox models with time to first depression insurance declaration or first antidepressant prescription as the underlying timescale, when the number of exposed cases was sufficient ($m \geq 3$), adjusted for sex (both sexes only), age, first year of the farm's establishment, and the number of working years. Results for females and males were obtained with subgroup analysis (one separate model for each sex).

Table S5: Agricultural activities and risks of depression, TRACTOR, France, 2002-2016 - results from the sensitivity analysis 2

Activity	Both sexes (n=1088561)					Female (n=337263)					Male (n=751298)				
	n (%)	m (range)	HR	p	p.adj	n (%)	m (range)	HR	p	p.adj	n (%)	m (range)	HR	p	p.adj
Truck farming, floriculture/ flower-growing	43487 (3·99)	177; 3336	1·13 [1·06-1·20]	0·05	0·08	13251 (3·93)	77; 1385	1·08 [0·83-1·43]	0·11	0·24	30236 (4·02)	100; 1951	1·14 [1·05-1·24]	0·03	0·06
Fruit arboriculture	25001 (2·3)	103; 1743	0·95 [0·88-1·02]	0·25	0·31	7927 (2·35)	51; 759	1·00 [0·62-1·62]	0·29	0·48	17074 (2·27)	52; 984	0·93 [0·79-1·39]	0·26	0·29
Garden center/tree nursery	5380 (0·49)	21; 450	1·09 [0·93-1·28]	0·36	0·41	1419 (0·42)	7; 154	1·34 [0·73-2·54]	0·36	0·48	3961 (0·53)	14; 296	1·06 [0·97-1·43]	0·06	0·09
Crop farming (e.g., wheat, corn, and industrial grower)	316926 (29·1)	1309; 22822	1·10 [1·08-1·13]	0·03	0·07	105642 (31·3)	719; 10953	1·05 [0·89-1·25]	0·14	0·28	211284 (28·1)	590; 11869	1·06 [1·03-1·10]	0·03	0·06
Viticulture	121950 (11·2)	489; 10497	**1·28 [1·21-1·34]**	3·2e-3	**0·01**	42909 (12·7)	240; 4918	1·01 [0·79-1·28]	0·27	0·48	79041 (10·5)	249; 5579	**1·15 [1·08-1·24]**	1·6e-3	**0·01**
Sylviculture/forestry (e.g., thinning, pruning)	2160 (0·2)	12; 145	1·15 [0·90-1·46]	0·19	0·25	359 (0·11)	8; 45	1·75 [0·68-4·49]	0·61	0·63	1801 (0·24)	4; 100	1·11 [0·79-1·58]	0·31	0·34
Unspecified specialized farming (e.g., herbs, mushrooms)	6615 (0·61)	31; 422	1·04 [0·89-1·20]	0·45	0·49	2408 (0·71)	16; 181	0·90 [0·40-2·14]	0·43	0·49	4207 (0·56)	15; 241	1·14 [0·92-1·41]	0·16	0·21
Dairy farming	161436 (14·8)	1009; 15000	**1·37 [1·33-1·41]**	< 0·0001	**< 0·0001**	49659 (14·7)	486; 6669	**1·40 [1·26-1·56]**	< 0·0001	**6·0e-4**	111777 (14·9)	523; 8331	**1·29 [1·24-1·34]**	< 0·0001	**1·0e-3**
Cow farming	111873 (10·3)	780; 10327	**1·17 [1·14-1·21]**	1·2e-4	**6·4e-4**	32904 (9·76)	386; 4521	**1·13 [1·08-1·32]**	1·2e-3	**0·01**	78969 (10·5)	394; 5806	**1·16 [1·11-1·21]**	3·2e-3	**0·01**
Both/mixed dairy and cow farming	31070 (2·85)	175; 2837	**1·14 [1·08-1·19]**	1·3e-3	**4·8e-3**	8084 (2·4)	73; 1103	**1·11 [1·06-1·49]**	3·5e-3	**0·02**	22986 (3·06)	102; 1734	**1·14 [1·09-1·26]**	6·7e-3	**0·02**
Ovine and caprine farming	48716 (4·48)	267; 3545	1·02 [0·97-1·07]	0·06	0·10	17314 (5·13)	131; 1742	0·90 [0·59-1·37]	0·35	0·48	31402 (4·18)	136; 1803	1·04 [0·97-1·12]	0·06	0·09
Pig farming	13636 (1·25)	72; 1239	**1·15 [1·05-1·25]**	0·03	**0·05**	3890 (1·15)	40; 530	0·79 [0·54-1·19]	0·39	0·48	9746 (1·3)	32; 709	**1·18 [1·06-1·31]**	0·02	**0·05**
Stud farming	17164 (1·58)	50; 920	**0·74 [0·65-0·83]**	< 0·0001	**4·8e-4**	7408 (2·2)	33; 531	0·70 [0·42-1·09]	0·08	0·19	9756 (1·3)	17; 389	**0·75 [0·63-0·90]**	2·5e-3	**0·01**
Training, dressage, riding clubs	14874 (1·37)	29; 969	0·79 [0·69-0·91]	0·03	0·06	6619 (1·96)	13; 516	0·64 [0·37-1·05]	0·06	0·16	8255 (1·1)	16; 453	**0·78 [0·66-0·95]**	9·6e-3	**0·03**
Unspecified large animal farming (e.g., ostrich, llama)	2997 (0·28)	8; 208	1·26 [1·01-1·58]	0·05	0·08	1437 (0·43)	5; 122	**1·28 [1·09-1·65]**	9·8e-3	**0·05**	1560 (0·21)	3; 86	1·21 [0·88-1·86]	0·23	0·27
Poultry and rabbit farming	25540 (2·35)	148; 2391	**1·26 [1·18-1·35]**	3·5e-4	**1·5e-3**	9995 (2·96)	77; 1247	1·47 [1·01-2·66]	0·04	0·12	15545 (2·07)	71; 1144	**1·23 [1·12-1·34]**	1·4e-3	**0·01**
Unspecified small animal farming (e.g., frogs, snails, bees)	19896 (1·83)	56; 897	**1·53 [1·42-1·65]**	< 0·0001	**< 0·0001**	8596 (2·55)	23; 416	1·38 [0·57-3·44]	0·34	0·48	11300 (1·5)	33; 481	1·14 [0·97-1·40]	0·06	0·09
Shellfish farming (e.g. oyster farming, scallop aquaculture)	3795 (0·35)	10; 251	1·12 [0·91-1·40]	0·15	0·22	725 (0·22)	3; 70	1·34 [0·69-2·51]	0·45	0·49	3070 (0·41)	7; 181	1·25 [0·97-1·64]	0·07	0·10
Unspecified and mixed farming (e.g., polyculture, mixed farming, diversified farming)	127900 (11·7)	604; 10223	0·99 [0·95-1·02]	0·18	0·25	39338 (11·7)	289; 4456	1·25 [1·02-2·32]	0·03	0·10	88562 (11·8)	315; 5767	1·01 [0·94-1·05]	0·08	0·11
Salt works/salt evaporation pond	974 (0·09)	2; 50	0·97 [0·63-1·52]	0·51	0·53	221 (22·7)	0; 9	1·07 [0·65-1·77]	0·39	0·48	753 (0·1)	2; 41	0·87 [0·53-1·33]	0·35	0·36
Wood production (e.g., lopping)	11385 (1·05)	27; 721	1·05 [0·91-1·21]	0·26	0·31	297 (0·09)	2; 35	1·13 [0·56-2·37]	0·42	0·49	11088 (1·48)	25; 686	1·05 [0·90-1·23]	0·19	0·24
Stationary sawmill (e.g., edging, trimming, decking, debarking)	787 (0·07)	3; 61	1·16 [0·77-1·82]	0·62	0·62	52 (0·02)	0; 9	1·46 [1·04-6·23]	0·02	0·09	735 (0·1)	3; 52	1·07 [0·67-1·78]	0·37	0·37
Agricultural work companies (e.g., pesticide applications, harvest reaping)	15557 (1·43)	51; 1081	1·25 [1·11-1·40]	0·02	0·06	1866 (0·55)	14; 199	0·92 [0·57-1·51]	0·38	0·48	13691 (1·82)	37; 882	1·19 [1·05-1·36]	0·03	0·06
Gardening, landscaping and reforestation companies	48878 (4·49)	120; 3036	1·12 [1·03-1·23]	0·04	0·07	2531 (0·75)	16; 238	1·21 [1·04-1·63]	0·03	0·10	46347 (6·17)	104; 2798	**1·17 [1·07-1·28]**	0·01	**0·03**
Company representative/ authorized representative	1931 (0·18)	9; 148	**1·64 [1·29-2·12]**	6·7e-3	**0·02**	1496 (0·44)	7; 122	1·47 [0·75-3·87]	0·78	0·78	437 (0·06)	2; 26	**2·08 [1·19-3·67]**	8·4e-3	**0·03**
Rural craftsperson (e.g., mason, mechanics)	7701 (0·71)	0; 28	**0·05 [0·03-0·10]**	< 0·0001	**< 0·0001**	283 (0·08)	0	NC	NC	NC	7418 (0·99)	0; 24	**0·04 [0·02-0·08]**	< 0·0001	**< 0·0001**

Note: HR: hazard ratio, n: number of exposed farm managers, m: range of the number of exposed depression cases, NC: not calculated, p: p-value, p.adj: p-value adjusted using the Benjamini-Hochberg approach. Hazard ratios were estimated by Cox models with time to first depression insurance declaration or first antidepressant prescription as the underlying timescale, when the number of exposed cases was sufficient ($m \geq 3$), adjusted for sex (both sexes only), age, first year of the farm's establishment, the number of working years, and the number of pre-existing comorbidities. Results for females and males were obtained with subgroup analysis (one separate model for each sex).

Table S6: Agricultural activities and risks of depression, TRACTOR, France, 2002-2016 - results from the sensitivity analysis 3

**Activity**	**Both sexes** (n=1088561)					**Female** (n=337263)					**Male** (n=751298)				
	**n (%)**	**m (range)**	**HR**	**p**	**p.adj**	**n (%)**	**m (range)**	**HR**	**p**	**p.adj**	**n (%)**	**m (range)**	**HR**	**p**	**p.adj**
Truck farming, floriculture/ flower-growing	43487 (3·99)	80; 1855	**0·75 [0·69-0·81]**	< 0·0001	**< 0·0001**	13251 (3·93)	26; 728	**0·87 [0·78-0·97]**	0·02	**0·03**	30236 (4·02)	54; 1127	**0·67 [0·61-0·75]**	3·3e-3	**3·3e-3**
Fruit arboriculture	25001 (2·3)	51; 1027	0·93 [0·84-1·03]	0·20	0·32	7927 (2·35)	21; 405	0·83 [0·71-0·99]	0·08	0·10	17074 (2·27)	30; 622	1·00 [0·88-1·14]	0·81	0·99
Garden center/tree nursery	5380 (0·49)	11; 282	1·24 [1·02-1·53]	0·10	0·12	1419 (0·42)	4; 95	1·32 [0·96-1·85]	0·16	0·19	3961 (0·53)	7; 187	1·20 [0·94-1·56]	0·20	0·28
Crop farming (e.g., wheat, corn, and industrial grower)	316926 (29·1)	547; 10755	**0·82 [0·80-0·85]**	< 0·0001	**< 0·0001**	105642 (31·3)	261; 4272	**0·76 [0·72-0·81]**	< 0·0001	**< 0·0001**	211284 (28·1)	286; 6483	**0·87 [0·84-0·91]**	< 0·0001	**< 0·0001**
Viticulture	121950 (11·2)	266; 6531	1·02 [0·98-1·06]	0·41	0·54	42909 (12·7)	112; 2825	0·99 [0·94-1·05]	0·56	0·79	79041 (10·5)	154; 3706	1·05 [0·99-1·11]	0·13	0·17
Sylviculture/forestry (e.g., thinning, pruning)	2160 (0·2)	6; 80	1·37 [0·97-1·98]	0·12	0·17	359 (0·11)	4; 19	1·18 [0·64-2·21]	0·59	0·92	1801 (0·24)	2; 61	1·50 [1·01-2·22]	0·09	0·11
Unspecified specialized farming (e.g., herbs, mushrooms)	6615 (0·61)	22; 271	**1·32 [1·10-1·57]**	4·4e-3	**6·3e-3**	2408 (0·71)	11; 122	1·28 [0·99-1·67]	0·08	0·12	4207 (0·56)	11; 149	**1·34 [1·04-1·73]**	0·03	**0·05**
Dairy farming	161436 (14·8)	545; 9135	**1·27 [1·23-1·32]**	< 0·0001	**< 0·0001**	49659 (14·7)	228; 3615	**1·33 [1·26-1·41]**	< 0·0001	**< 0·0001**	111777 (14·9)	317; 5520	**1·22 [1·16-1·28]**	< 0·0001	**< 0·0001**
Cow farming	111873 (10·3)	481; 6933	**1·41 [1·36-1·47]**	< 0·0001	**< 0·0001**	32904 (9·76)	203; 2840	**1·48 [1·40-1·56]**	< 0·0001	**< 0·0001**	78969 (10·5)	278; 4093	**1·34 [1·27-1·40]**	< 0·0001	**< 0·0001**
Both/mixed dairy and cow farming	31070 (2·85)	98; 1834	**1·10 [1·02-1·19]**	0·03	**0·04**	8084 (2·4)	37; 646	**1·19 [1·05-1·34]**	0·03	**0·03**	22986 (3·06)	61; 1188	1·04 [0·94-1·15]	0·47	0·78
Ovine and caprine farming	48716 (4·48)	150; 2327	1·04 [0·98-1·11]	0·23	0·34	17314 (5·13)	70; 1093	1·04 [0·95-1·15]	0·44	0·64	31402 (4·18)	80; 1234	1·04 [0·96-1·14]	0·40	0·58
Pig farming	13636 (1·25)	42; 845	**1·17 [1·04-1·31]**	0·04	**0·04**	3890 (1·15)	20; 345	**1·35 [1·14-1·61]**	6·6e-3	**6·9e-3**	9746 (1·3)	22; 500	1·05 [0·91-1·22]	0·43	0·69
Stud farming	17164 (1·58)	33; 671	1·05 [0·91-1·21]	0·57	0·74	7408 (2·2)	20; 402	1·02 [0·85-1·22]	0·52	0·71	9756 (1·3)	13; 269	1·11 [0·90-1·40]	0·43	0·57
Training, dressage, riding clubs	14874 (1·37)	19; 742	0·98 [0·84-1·15]	0·39	0·49	6619 (1·96)	9; 411	1·05 [0·87-1·29]	0·42	0·51	8255 (1·1)	10; 331	0·93 [0·73-1·19]	0·43	0·59
Unspecified large animal farming (e.g., ostrich, llama)	2997 (0·28)	5; 149	**1·95 [1·52-2·51]**	0·02	**0·02**	1437 (0·43)	3; 93	**1·90 [1·39-2·64]**	0·02	**0·02**	1560 (0·21)	2; 56	**1·88 [1·28-2·80]**	0·04	**0·05**
Poultry and rabbit farming	25540 (2·35)	81; 1620	**1·29 [1·19-1·41]**	1·5e-3	**1·5e-3**	9995 (2·96)	39; 824	**1·31 [1·17-1·47]**	0·01	**0·01**	15545 (2·07)	42; 796	**1·28 [1·14-1·44]**	5·4e-3	**5·8e-3**
Unspecified small animal farming (e.g., frogs, snails, bees)	19896 (1·83)	31; 614	0·88 [0·76-1·02]	0·18	0·22	8596 (2·55)	10; 277	**0·65 [0·52-0·82]**	7·3e-3	**7·7e-3**	11300 (1·5)	21; 337	1·14 [0·95-1·36]	0·23	0·32
Shellfish farming (e.g. oyster farming, scallop aquaculture)	3795 (0·35)	7; 159	**1·43 [1·10-1·89]**	0·05	**0·05**	725 (0·22)	2; 45	1·29 [0·85-1·98]	0·29	0·43	3070 (0·41)	5; 114	**1·55 [1·13-2·16]**	0·04	**0·05**
Unspecified and mixed farming (e.g., polyculture, mixed farming, diversified farming)	127900 (11·7)	346; 6690	**1·20 [1·15-1·24]**	2·8e-3	**2·8e-3**	39338 (11·7)	144; 2679	**1·26 [1·19-1·34]**	5·1e-3	**5·1e-3**	88562 (11·8)	202; 4011	**1·13 [1·07-1·20]**	0·02	**0·02**
Salt works/salt evaporation pond	974 (0·09)	2; 40	0·98 [0·61-1·60]	0·80	0·91	221 (22·7)	0; 3	0·43 [0·17-1·10]	0·08	0·11	753 (0·1)	2; 34	1·36 [0·82-2·25]	0·25	0·40
Wood production (e.g., lopping)	11385 (1·05)	13; 504	1·12 [0·94-1·34]	0·21	0·28	297 (0·09)	0; 22	**2·21 [1·22-4·02]**	0·01	**0·02**	11088 (1·48)	13; 482	1·10 [0·92-1·33]	0·31	0·40
Stationary sawmill (e.g., edging, trimming, decking, debarking)	787 (0·07)	3; 45	1·62 [1·01-2·71]	0·14	0·18	52 (0·02)	0; 52	**3·64 [1·43-9·60]**	0·02	**0·03**	735 (0·1)	3; 37	1·38 [0·82-2·40]	0·34	0·45
Agricultural work companies (e.g., pesticide applications, harvest reaping)	15557 (1·43)	20; 656	1·15 [0·99-1·35]	0·12	0·15	1866 (0·55)	3; 107	**1·52 [1·12-2·09]**	0·04	**0·04**	13691 (1·82)	17; 549	1·10 [0·93-1·32]	0·34	0·42
Gardening, landscaping and reforestation companies	48878 (4·49)	76; 2309	**1·36 [1·24-1·49]**	7·8e-3	**7·8e-3**	2531 (0·75)	9; 169	**1·87 [1·42-2·49]**	3·3e-3	**3·3e-3**	46347 (6·17)	67; 2140	**1·37 [1·25-1·51]**	0·01	**0·01**
Company representative/ authorized representative	1931 (0·18)	2; 42	0·86 [0·57-1·31]	0·49	0·74	1496 (0·44)	1; 35	0·80 [0·51-1·27]	0·35	0·55	437 (0·06)	1; 7	1·17 [0·42-3·36]	0·73	0·99
Rural craftsperson (e.g., mason, mechanics)	7701 (0·71)	0; 5	**0·03 [0·01-0·07]**	< 0·0001	**< 0·0001**	283 (0·08)	0	NC	NC	NC	7418 (0·99)	0; 5	**0·03 [0·01-0·07]**	< 0·0001	**< 0·0001**

Note: HR: hazard ratio, n: number of exposed farm managers, m: range of the number of exposed depression cases, NC: not calculated, p: p-value, p.adj: p-value adjusted using the Benjamini-Hochberg approach. Hazard ratios were estimated by Cox models with time to first depression insurance declaration or first antidepressant prescription as the underlying timescale, when the number of exposed cases was sufficient ($m \geq 3$), adjusted for sex (both sexes only), age, first year of the farm's establishment, the number of working years, the number of pre-existing comorbidities, and on other potential covariates (Table 1). Results for females and males were obtained with subgroup analysis (one separate model for each sex).

Table S7: Agricultural activities and risks of depression, TRACTOR, France, 2002-2016 - results from the sensitivity analysis 4 restricted to SSRIs

**Activity**	**Both sexes** (n=1088561)					**Female** (n=337263)					**Male** (n=751298)				
	**n (%)**	**m (range)**	**HR**	**p**	**p.adj**	**n (%)**	**m (range)**	**HR**	**p**	**p.adj**	**n (%)**	**m (range)**	**HR**	**p**	**p.adj**
Truck farming, floriculture/ flower-growing	43487 (3·99)	80; 1855	1·08 [0·98-1·18]	0·15	0·22	13251 (3·93)	26; 728	1·11 [0·98-1·27]	0·15	0·19	30236 (4·02)	54; 1127	1·05 [0·92-1·21]	0·51	0·63
Fruit arboriculture	25001 (2·3)	51; 1027	1·00 [0·88-1·13]	0·67	0·94	7927 (2·35)	21; 405	0·87 [0·71-1·06]	0·21	0·30	17074 (2·27)	30; 622	1·09 [0·93-1·29]	0·28	0·43
Garden center/tree nursery	5380 (0·49)	11; 282	1·25 [0·98-1·63]	0·13	0·16	1419 (0·42)	4; 95	1·39 [0·94-2·10]	0·12	0·17	3961 (0·53)	7; 187	1·17 [0·84-1·66]	0·42	0·60
Crop farming (e.g., wheat, corn, and industrial grower)	316926 (29·1)	547; 10755	**0·89 [0·85-0·93]**	4·4e-4	**4·5e-4**	105642 (31·3)	261; 4272	**0·88 [0·83-0·94]**	0·03	**0·03**	211284 (28·1)	286; 6483	**0·93 [0·88-0·98]**	0·02	**0·03**
Viticulture	121950 (11·2)	266; 6531	**1·10 [1·05-1·16]**	1·5e-3	**1·6e-3**	42909 (12·7)	112; 2825	1·05 [0·98-1·13]	0·20	0·27	79041 (10·5)	154; 3706	**1·16 [1·08-1·24]**	0·02	**0·02**
Sylviculture/forestry (e.g., thinning, pruning)	2160 (0·2)	6; 80	1·46 [0·95-2·25]	0·13	0·18	359 (0·11)	4; 19	1·91 [0·99-3·71]	0·07	0·10	1801 (0·24)	2; 61	1·40 [0·82-2·41]	0·29	0·41
Unspecified specialized farming (e.g., herbs, mushrooms)	6615 (0·61)	22; 271	**1·45 [1·17-1·80]**	1·1e-3	**1·5e-3**	2408 (0·71)	11; 122	1·39 [1·03-1·89]	0·05	0·06	4207 (0·56)	11; 149	**1·45 [1·06-1·99]**	0·03	**0·04**
Dairy farming	161436 (14·8)	545; 9135	**1·38 [1·32-1·44]**	< 0·0001	**< 0·0001**	49659 (14·7)	228; 3615	**1·45 [1·36-1·56]**	< 0·0001	**< 0·0001**	111777 (14·9)	317; 5520	**1·24 [1·17-1·32]**	< 0·0001	**< 0·0001**
Cow farming	111873 (10·3)	481; 6933	**1·53 [1·46-1·61]**	< 0·0001	**< 0·0001**	32904 (9·76)	203; 2840	**1·61 [1·50-1·73]**	< 0·0001	**< 0·0001**	78969 (10·5)	278; 4093	**1·44 [1·35-1·54]**	< 0·0001	**< 0·0001**
Both/mixed dairy and cow farming	31070 (2·85)	98; 1834	**1·20 [1·09-1·32]**	8·9e-4	**9·3e-4**	8084 (2·4)	37; 646	**1·28 [1·11-1·50]**	4·1e-3	**4·5e-3**	22986 (3·06)	61; 1188	1·12 [0·99-1·26]	0·08	0·12
Ovine and caprine farming	48716 (4·48)	150; 2327	**1·14 [1·05-1·22]**	2·0e-3	**2·5e-3**	17314 (5·13)	70; 1093	1·12 [1·00-1·25]	0·07	0·09	31402 (4·18)	80; 1234	**1·15 [1·03-1·28]**	0·02	**0·03**
Pig farming	13636 (1·25)	42; 845	**1·28 [1·11-1·47]**	8·6e-3	**8·9e-3**	3890 (1·15)	20; 345	**1·40 [1·14-1·72]**	2·8e-3	**3·5e-3**	9746 (1·3)	22; 500	1·17 [0·98-1·40]	0·14	0·18
Stud farming	17164 (1·58)	33; 671	1·16 [0·98-1·37]	0·14	0·18	7408 (2·2)	20; 402	1·02 [0·83-1·27]	0·60	0·84	9756 (1·3)	13; 269	**1·37 [1·07-1·76]**	0·04	**0·04**
Training, dressage, riding clubs	14874 (1·37)	19; 742	1·03 [0·86-1·24]	0·67	0·82	6619 (1·96)	9; 411	1·02 [0·81-1·29]	0·58	0·77	8255 (1·1)	10; 331	1·02 [0·78-1·37]	0·68	0·87
Unspecified large animal farming (e.g., ostrich, llama)	2997 (0·28)	5; 149	**1·80 [1·30-2·54]**	0·03	**0·03**	1437 (0·43)	3; 93	1·68 [1·14-2·52]	0·05	0·06	1560 (0·21)	2; 56	1·66 [0·95-2·96]	0·19	0·23
Poultry and rabbit farming	25540 (2·35)	81; 1620	**1·42 [1·29-1·57]**	< 0·0001	**< 0·0001**	9995 (2·96)	39; 824	**1·47 [1·29-1·69]**	3·9e-3	**3·9e-3**	15545 (2·07)	42; 796	**1·35 [1·17-1·57]**	2·7e-3	**3·1e-3**
Unspecified small animal farming (e.g., frogs, snails, bees)	19896 (1·83)	31; 614	0·89 [0·76-1·06]	0·25	0·36	8596 (2·55)	10; 277	**0·61 [0·47-0·79]**	4·1e-3	**4·1e-3**	11300 (1·5)	21; 337	1·25 [1·01-1·55]	0·06	0·08
Shellfish farming (e.g. oyster farming, scallop aquaculture)	3795 (0·35)	7; 159	1·40 [1·01-1·95]	0·06	0·08	725 (0·22)	2; 45	1·35 [0·80-2·27]	0·28	0·44	3070 (0·41)	5; 114	**1·61 [1·08-2·44]**	0·04	**0·04**
Unspecified and mixed farming (e.g., polyculture, mixed farming, diversified farming)	127900 (11·7)	346; 6690	**1·29 [1·22-1·37]**	< 0·0001	**< 0·0001**	39338 (11·7)	144; 2679	**1·33 [1·23-1·44]**	< 0·0001	**< 0·0001**	88562 (11·8)	202; 4011	**1·22 [1·13-1·31]**	2·0e-4	**2·0e-4**
Salt works/salt evaporation pond	974 (0·09)	2; 40	1·22 [0·72-2·08]	0·49	0·69	221 (22·7)	0; 3	0·49 [0·18-1·34]	0·16	0·18	753 (0·1)	2; 34	1·87 [1·07-3·30]	0·03	0·06
Wood production (e.g., lopping)	11385 (1·05)	13; 504	1·05 [0·84-1·32]	0·61	0·84	297 (0·09)	0; 22	1·91 [0·88-4·21]	0·14	0·21	11088 (1·48)	13; 482	1·10 [0·87-1·40]	0·41	0·59
Stationary sawmill (e.g., edging, trimming, decking, debarking)	787 (0·07)	3; 45	1·70 [0·97-3·14]	0·15	0·19	52 (0·02)	0; 52	**4·63 [1·81-12·1]**	5·3e-3	**6·2e-3**	735 (0·1)	3; 37	1·39 [0·72-2·79]	0·43	0·57
Agricultural work companies (e.g., pesticide applications, harvest reaping)	15557 (1·43)	20; 656	1·14 [0·94-1·38]	0·27	0·35	1866 (0·55)	3; 107	1·16 [0·76-1·83]	0·51	0·75	13691 (1·82)	17; 549	1·19 [0·97-1·47]	0·21	0·25
Gardening, landscaping and reforestation companies	48878 (4·49)	76; 2309	**1·32 [1·19-1·48]**	0·01	**0·01**	2531 (0·75)	9; 169	**1·72 [1·27-2·37]**	0·01	**0·01**	46347 (6·17)	67; 2140	**1·50 [1·34-1·69]**	2·5e-3	**2·5e-3**
Company representative/ authorized representative	1931 (0·18)	2; 42	1·01 [0·62-1·67]	0·71	0·96	1496 (0·44)	1; 35	1·00 [0·60-1·67]	0·73	0·98	437 (0·06)	1; 7	1·34 [0·68-3·84]	0·55	0·67
Rural craftsperson (e.g., mason, mechanics)	7701 (0·71)	0; 5	**0·03 [0·01-0·09]**	< 0·0001	**< 0·0001**	283 (0·08)	0	NC	NC	NC	7418 (0·99)	0; 5	**0·03 [0·01-0·10]**	< 0·0001	**< 0·0001**

Note: HR: hazard ratio, n: number of exposed farm managers, m: range of the number of exposed depression cases, NC: not calculated, p: p-value, p.adj: p-value adjusted using the Benjamini-Hochberg approach. Hazard ratios were estimated by Cox models with time to first depression insurance declaration or first antidepressant prescription as the underlying timescale, when the number of exposed cases was sufficient ($m \geq 3$), adjusted for sex (both sexes only), age, first year of the farm's establishment, the number of working years, and the number of pre-existing comorbidities. Results for females and males were obtained with subgroup analysis (one separate model for each sex).

STROBE Statement Checklist

	Item No	Recommendation	Page
**Title and abstract**	1	(*a*) Indicate the study's design with a commonly used term in the title or the abstract	1, 3, 4
		(*b*) Provide in the abstract an informative and balanced summary of what was done and what was found	3, 4
**Introduction**			
Background/rationale	2	Explain the scientific background and rationale for the investigation being reported	6
Objectives	3	State specific objectives, including any prespecified hypotheses	6
**Methods**			
Study design	4	Present key elements of study design early in the paper	7, 8
Setting	5	Describe the setting, locations, and relevant dates, including periods of recruitment, exposure, follow-up, and data collection	7, 8
Participants	6	(*a*) Give the eligibility criteria, and the sources and methods of selection of participants. Describe methods of follow-up	7, 8
		(*b*) For matched studies, give matching criteria and number of exposed and unexposed	DNA
Variables	7	Clearly define all outcomes, exposures, predictors, potential confounders, and effect modifiers. Give diagnostic criteria, if applicable	7-10, Table 1
Data sources/ measurement	8*	For each variable of interest, give sources of data and details of methods of assessment (measurement). Describe comparability of assessment methods if there is more than one group	7-10, Table 1
Bias	9	Describe any efforts to address potential sources of bias	7-10
Study size	10	Explain how the study size was arrived at	7
Quantitative variables	11	Explain how quantitative variables were handled in the analyses. If applicable, describe which groupings were chosen and why	8-10, Table 1
Statistical methods	12	(*a*) Describe all statistical methods, including those used to control for confounding	8-10
		(*b*) Describe any methods used to examine subgroups and interactions	8-10
		(*c*) Explain how missing data were addressed	7
		(*d*) If applicable, explain how loss to follow-up was addressed	DNA
		(*e*) Describe any sensitivity analyses	9, 10
**Results**			
Participants	13*	(a) Report numbers of individuals at each stage of study-e.g., numbers potentially eligible, examined for eligibility, confirmed eligible, included in the study, and analyzed	10, 11, Table 2
		(b) Give reasons for non-participation at each stage	DNA
		(c) Consider use of a flow diagram	DNA
Descriptive data	14*	(a) Give characteristics of study participants (e.g., demographic, clinical, social) and information on exposures and potential confounders	10, Table 2
		(b) Indicate number of participants with missing data for each variable of interest	10, Table 2
		(c) Summarize follow-up time (e.g., average and total amount)	10
Outcome data	15*	Report numbers of outcome events or summary measures over time	11, 12, Table 3
Main results	16	(*a*) Give unadjusted estimates and, if applicable, confounder-adjusted estimates and their precision (e.g., 95% confidence interval). Make clear which confounders were adjusted for and why they were included	Table 3, and Tables S3 to S7
		(*b*) Report category boundaries when continuous variables were categorized	DNA
		(*c*) If relevant, consider translating estimates of relative risk into absolute risk for a meaningful time period	DNA
Other analyses	17	Report other analyses done-e.g., analyses of subgroups and interactions, and sensitivity analyses	11-13, Table 3, and Tables S3 to S7
**Discussion**			
Key results	18	Summarize key results with reference to study objectives	13, 14
Limitations	19	Discuss limitations of the study, taking into account sources of potential bias or imprecision. Discuss both direction and magnitude of any potential bias	16-19
Interpretation	20	Give a cautious overall interpretation of results considering objectives, limitations, multiplicity of analyses, results from similar studies, and other relevant evidence	16-19
Generalizability	21	Discuss the generalizability (external validity) of the study results	18
**Other information**			
Funding	22	Give the source of funding and the role of the funders for the present study and, if applicable, for the original study on which the present article is based	4, 10, 20, 21

*Give information separately for exposed and unexposed groups.
DNA: does not applied.
