# Supplementary material for: Agricultural activities and risk of treatment for depressive disorders among the entire French agricultural workforce: the TRACTOR project, a nationwide retrospective cohort study
Source: Lancet Reg Health Eur. 2023 Jun 26;31:100674. doi: 10.1016/j.lanepe.2023.100674 (PMC10318497; doi:10.1016/j.lanepe.2023.100674)
Supplement: Supplementary Fig. S1 [file mmc3.pdf]

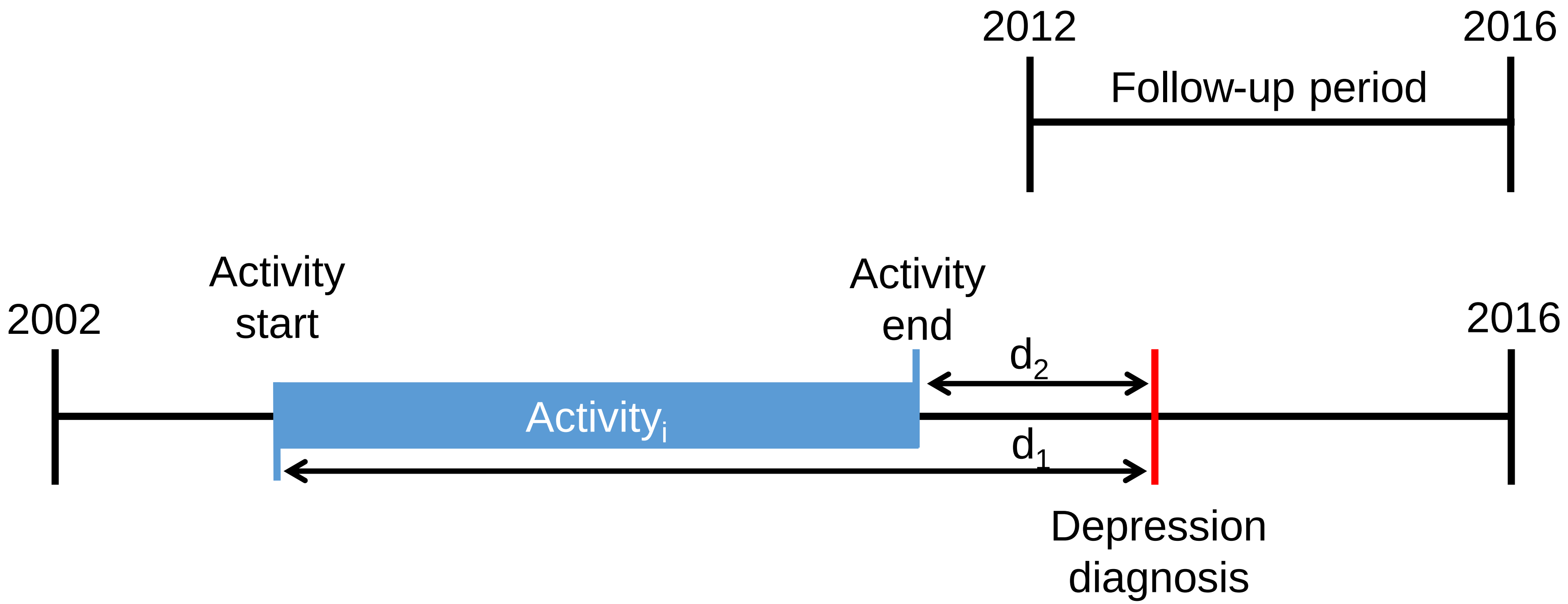

Depression considered as work-related if:

- $d_1 > 0$ year and $d_2 \leq 2$ years → for the main analysis, and sensitivity analyses 1, 3 and 4
- $d_1 > 0$ year → for sensitivity analysis 2
