# Supplementary figures and images for: Agricultural activities and risk of treatment for depressive disorders among the entire French agricultural workforce: the TRACTOR project, a nationwide retrospective cohort study
Source: Lancet Reg Health Eur. 2023 Jun 26;31:100674. doi: 10.1016/j.lanepe.2023.100674 (PMC10318497; doi:10.1016/j.lanepe.2023.100674)

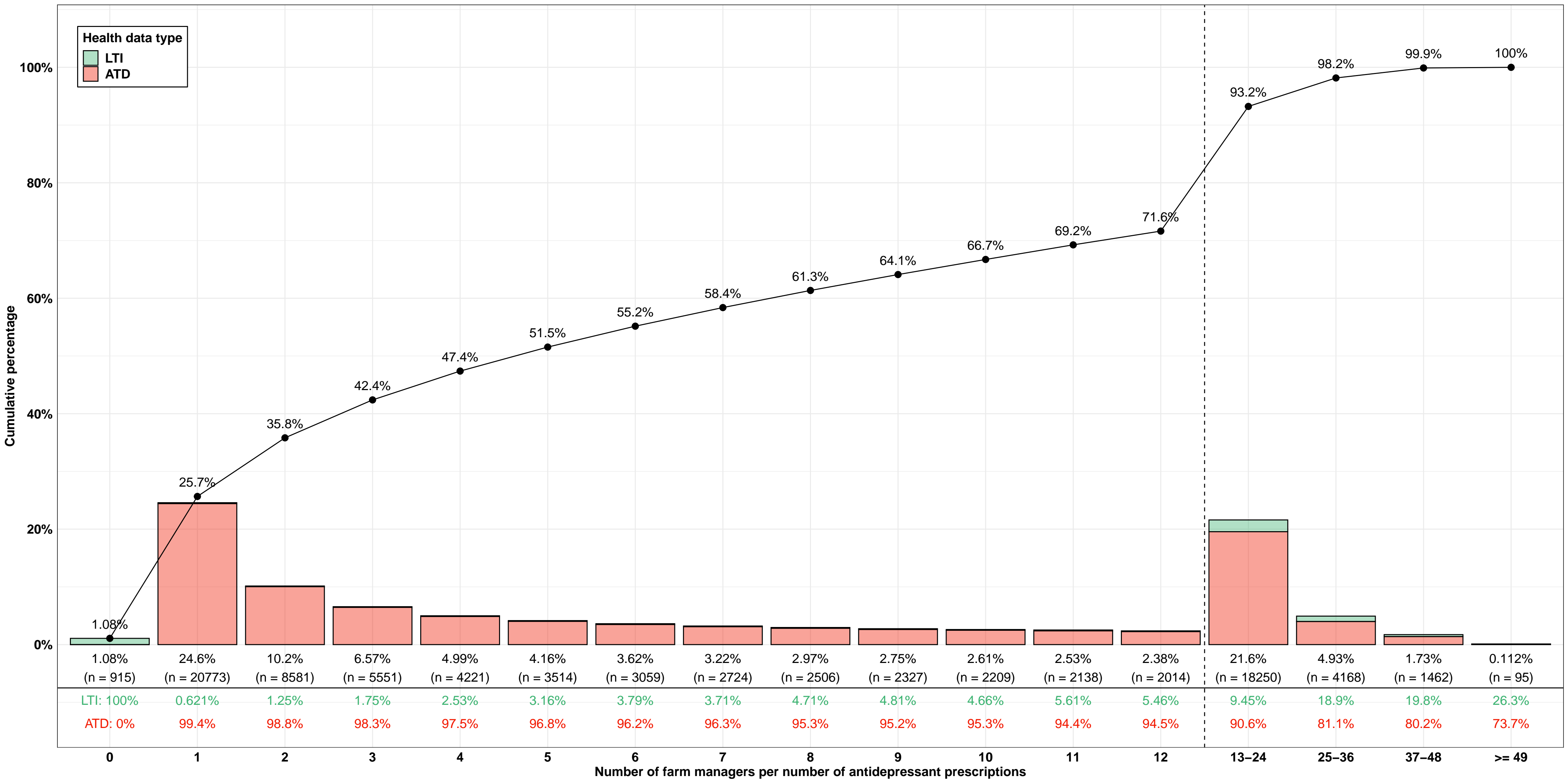

Supplement: Supplementary Fig. S2 [file mmc4.pdf]

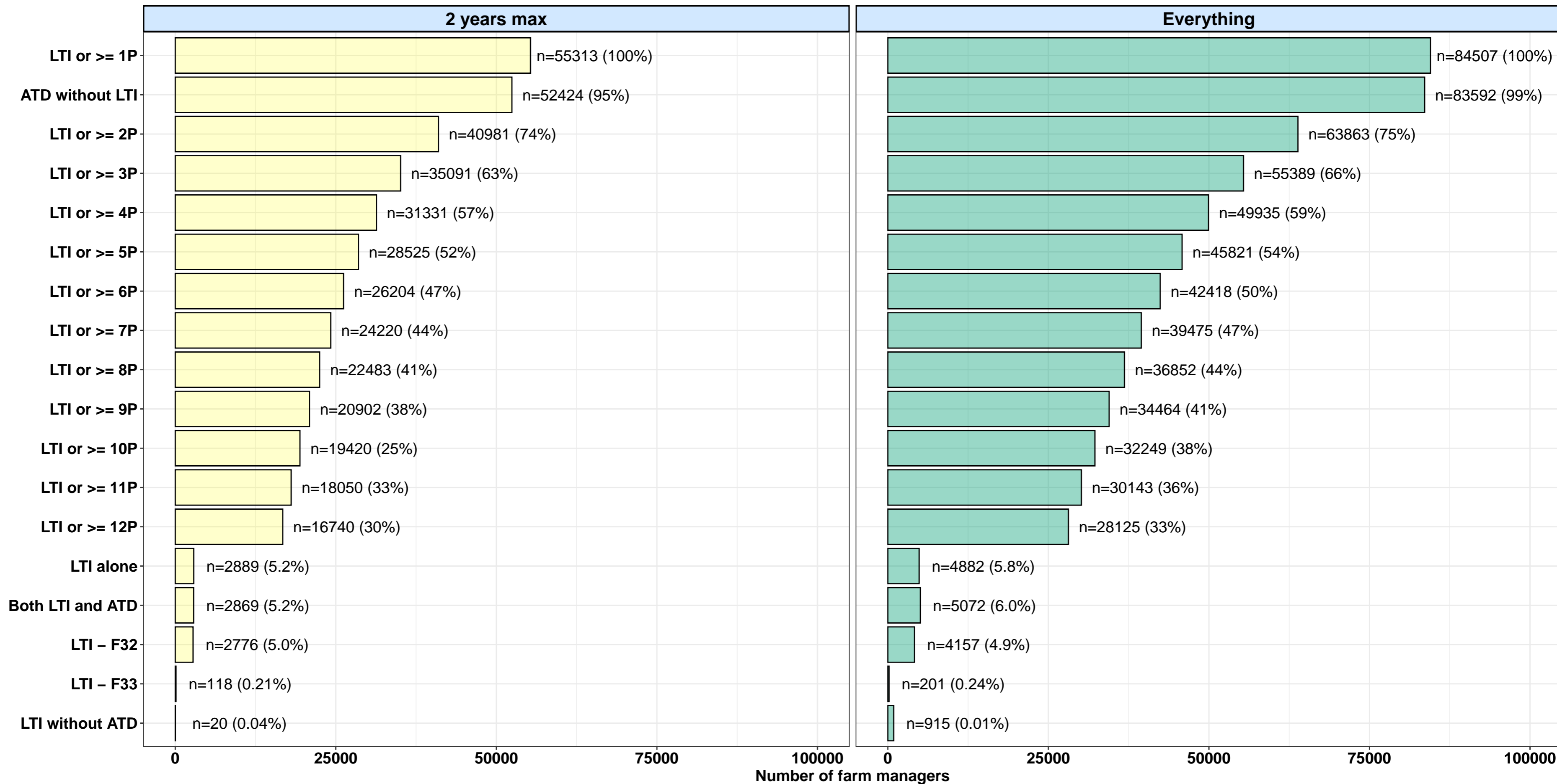

Supplement: Supplementary Fig. S3 [file mmc5.pdf]

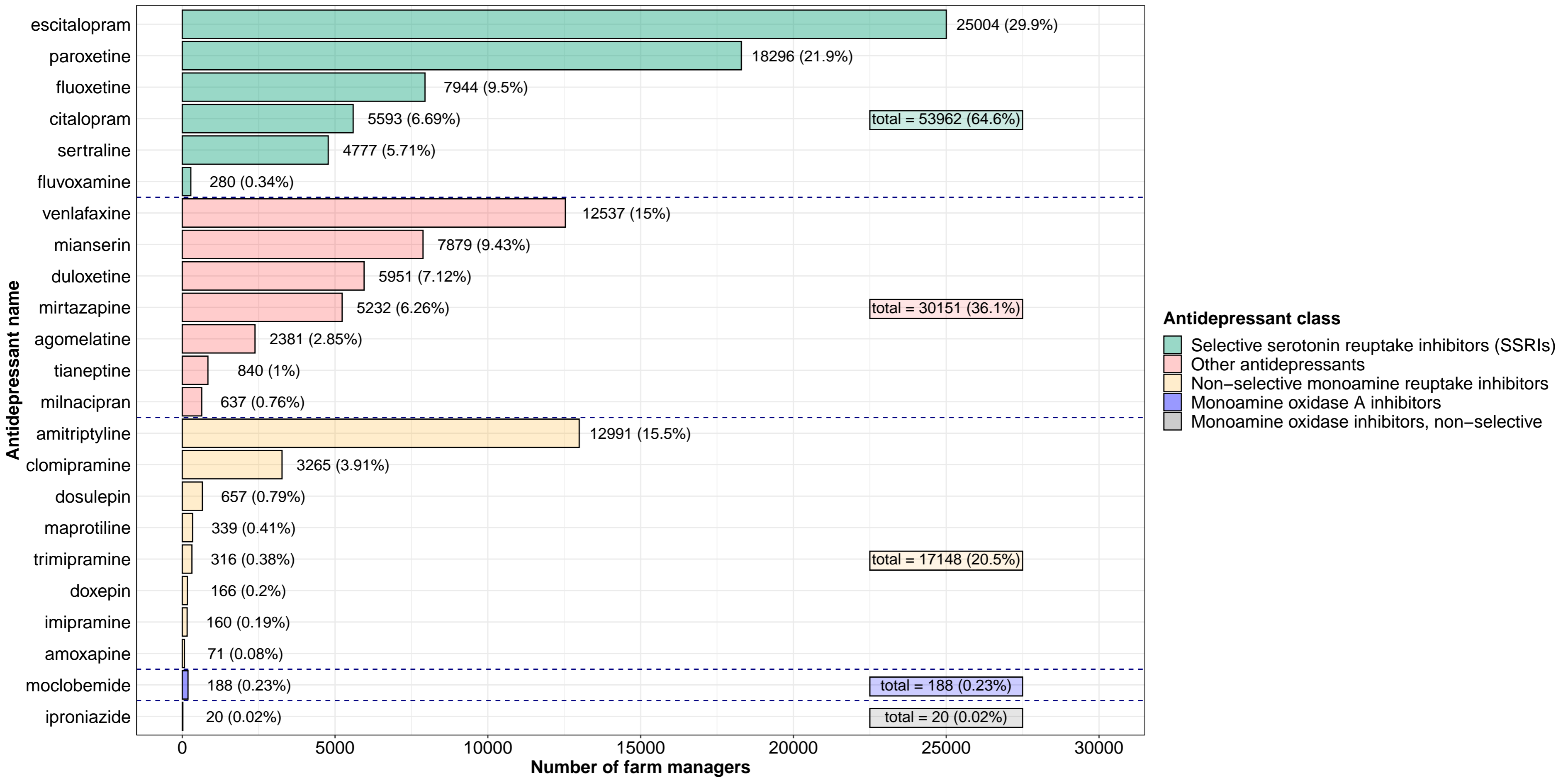

Supplement: Supplementary Fig. S4 [file mmc6.pdf]

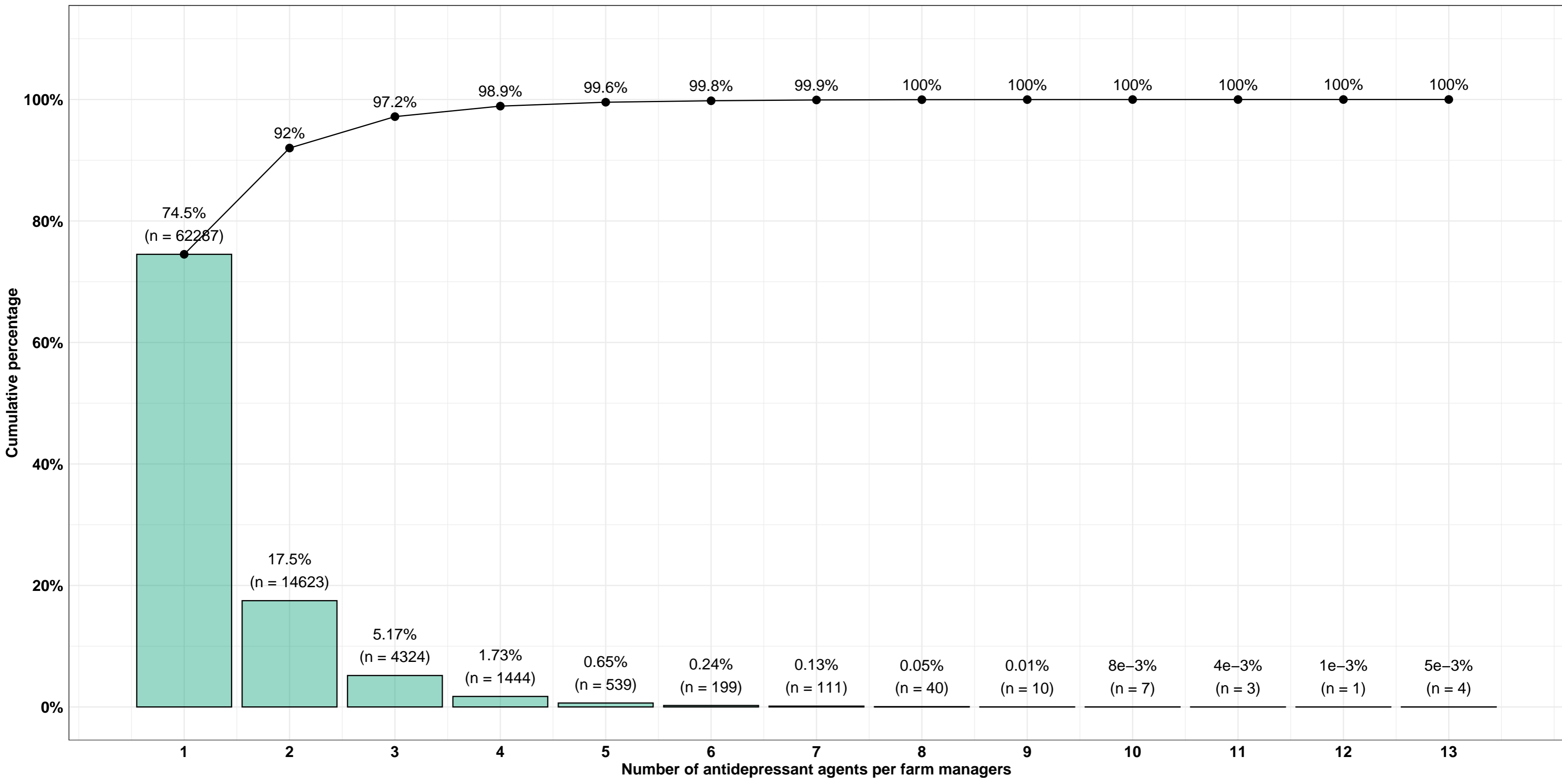

Supplement: Supplementary Fig. S5 [file mmc7.pdf]
