# Supplementary material for: Agricultural activities and risk of treatment for depressive disorders among the entire French agricultural workforce: the TRACTOR project, a nationwide retrospective cohort study
Source: Lancet Reg Health Eur. 2023 Jun 26;31:100674. doi: 10.1016/j.lanepe.2023.100674 (PMC10318497; doi:10.1016/j.lanepe.2023.100674)
Supplement: Translated abstract [file mmc8.docx]

*This translation in French was submitted by the authors and we reproduce it as supplied. It has not been peer reviewed. Our editorial processes have only been applied to the original abstract in English, which should serve as reference for this manuscript.*

**Translated abstract**

**Contexte**

Bien que la dépression soit un problème majeur au sein de la population agricole, il n'existe à ce jour que peu d’études portant sur des activités agricoles spécifiques. Notre objectif était de déterminer si, au sein de l’intégralité des non-salariés agricoles (NSA) français, certaines activités/pratiques agricoles étaient associées à un risque de dépression plus élevé que d’autre.

**Méthodes**

Cette étude (cohorte rétrospective à l’échelle nationale), a utilisé des données médico-administratives disponibles de la cadre du projet TRACTOR (détection et surveillance des risques professionnels au sein de l’ensemble de la population agricole française). Ces bases de données concernent l'ensemble de la population active agricole française travaillant en France métropolitaine. Les données ont été analysées de janvier 2021 à décembre 2022. Tous les NSA ayant travaillé au moins un an au cours de la période 2002-2016 ont été inclus dans l’étude. Des analyses de survie, avec le calcul de ratio de danger (RD), ont été conduites pour déterminer l’association statistique entre 26 activités agricoles et le risque de dépression. Ces analyses ont été ajustées sur l’âge, le sexe, l’année d’installation, et le nombre de comorbidités préexistantes. La date de première déclaration en affection longue durée/maladie professionnelle ou de première dispensation d’antidépresseur a été choisie comme échelle de temps. Pour chaque activité le groupe contrôle (de référence) comprenait NSA l’intégralité des NSA qui n’a pas effectué l’activité étudiée. Par exemple, lorsque l’élevage porcin était étudié, la population de référence correspondait à l’intégralité des NSA n’ayant pas fait d’élevage porcin sur la période d’étude. Quatre analyses de sensibilité ont été réalisés afin de tester des hypothèses, et d’étudier de potentielles sources de biais.

**Résultats**

Un total de 84507 cas de dépression traitée (7·76% ; 28·2 cas pour 1000 personnes-années) parmi 1088561 NSA ont été identifiés (âge moyen : 46·6 [SD 14·1]). Comparativement aux autres activités, l’élevage bovin-lait (RD=1·37, intervalle de confiance à 95%: 1·32-1·42), l’élevage bovin-viande (RD=1·53 [1·47-1·59]), l’élevage de volailles-lapins (RD=1·37 [1·27-1·50]), la polyculture/poly-élevage (RD=1·30 [1·24-1·36]) étaient associés à un risque de dépression plus élevé. Des différences entre sexes ont été observées, avec la plupart du temps, des risques de dépression plus élevés chez les femmes que chez les hommes.

**Interprétation**

Les activités agricoles les plus à risque de dépression ont été identifié au sein de l’intégralité de la population française des NSA. Ces résultats constituent une première étape cruciale pour définir les populations d’agriculteurs les plus à risques afin de cibler les mesures et actions de prévention. Les situations mises en évidence ont vocation à être interprétées collectivement, et peuvent appeler à la mise en place d’études ciblées pour mieux les préciser et/ou instaurer des mesures de prévention.

**Financement**

Ce travail a été financé par la MSA, et le MIAI@Grenoble Alpes.
